# Direct Kinetic Measurements of a Cyclic Criegee Intermediate; Unimolecular Decomposition of *c-*(CH_2_)_5_COO

**DOI:** 10.1021/acs.jpclett.4c00554

**Published:** 2024-05-10

**Authors:** Jari Peltola, Petri Heinonen, Arkke Eskola

**Affiliations:** Department of Chemistry, University of Helsinki, P.O. Box 55, A.I. Virtasen aukio 1, FI-00014 Helsinki, Finland

## Abstract

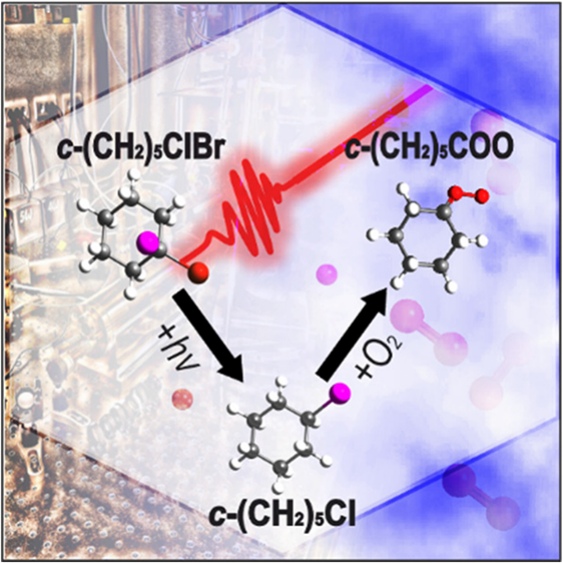

We report the first direct kinetic measurements of a cyclic stabilized Criegee Intermediate. We have measured the unimolecular reaction rate coefficient of cyclohexanone oxide (*c-*(CH_2_)_5_COO) in the temperature 213–296 K and pressure 7–50 Torr ranges using absorption spectrometry. The *c-*(CH_2_)_5_COO was produced by the photolysis of *c-*(CH_2_)_5_CIBr at 213 nm in the presence of O_2_. We compare the measured fast *c-*(CH_2_)_5_COO unimolecular rate coefficient, 1998 ± 147 s^–1^ at 296 K, with the literature calculations for the structurally similar *E*-nopinone oxide formed in β-pinene ozonolysis. The *k*_uni_(*c-*(CH_2_)_5_COO)/*k*_uni_(*E*-nopinone oxide) ratio calculated using transition-state theory and density functional theory agrees well with this comparison. We have also measured the bimolecular rate coefficient of the reaction between *c-*(CH_2_)_5_COO and trifluoroacetic acid at 253 K and 10 Torr and obtained the value (8.7 ± 1.0) × 10^–10^ cm^3^ molecule^–1^ s^–1^. This very large value agrees with previous kinetic measurements for reactions between stabilized Criegee intermediates and halogenated organic acids.

Many terpenes, e.g., monoterpenes and sesquiterpenes, are cyclic alkenes with one or more double bonds and play an important role in atmospheric chemistry. Terpenes are released into the troposphere from vegetation, especially from coniferous plants, with an estimated global emission rate on the order of 10^14^ g yr^–1^.^1,2^ The two most abundant monoterpenes in the troposphere are endocyclic α-pinene and exocyclic β-pinene. One important degradation process of alkenes in the atmosphere is the reaction with ozone, i.e. ozonolysis, where a highly excited primary ozonide is formed in a very exothermic O_3_ + alkene reaction.^3^ In the gas phase and at atmospheric pressure, any excited primary ozonide immediately decomposes to an excited carbonyl oxide, also known as a Criegee intermediate, and a stable carbonyl compound. Depending on the alkene and the reaction conditions in the gas phase, a significant fraction of the excited Criegee intermediate is thermalized, producing stabilized Criegee intermediate (sCI).

Uni- and bimolecular reactions of sCIs are sources of hydroxyl radicals (OH), acids, hydroperoxides, and aerosols in the troposphere.^4−8^ Unimolecular reaction of a *syn*-sCI, e.g. *c-*(CH_2_)_5_COO investigated in this work, leads to a labile vinylhydroperoxide that subsequently decomposes mainly to a resonantly stabilized vinoxy radical + OH.^5^ This unimolecular decomposition of sCIs is an important step in the chain-propagation arising from the atmospheric ozonolysis of terpenes, which can rapidly lead to highly oxygenated organic molecules (HOMs) with very-low volatilities.^9^ These recently discovered HOMs, formed via terpene autoxidation involving peroxyl radicals, contribute to the formation of secondary organic aerosols (SOAs), a major component of atmospheric aerosols known to affect the Earth’s radiation balance and adversely on human health.

So far, no direct kinetic measurement of a cyclic sCI has been performed successfully by any method. Since 2012, the method utilized to produce different alkyl-substituted sCIs in direct kinetic experiments has been to pulse-photolyze *gem*-diiodoalkane precursor (e.g., CH_2_I_2_) in the presence of O_2_.^5^ However, cyclic *gem*-diiodoalkane precursors are highly reactive and, according to the current understanding, cannot be prepared and purified.^10^ We have recently introduced new bromoiodoalkane-based precursors (R_1_R_2_CIBr, where R_1_, R_2_ is an alkyl group), whose photolysis at 213 nm in the presence of O_2_ produces the corresponding stabilized Criegee Intermediate, R_1_R_2_COO.^11,12^ These precursors have proven to be more stable than the corresponding *gem*-diiodoalkane compounds. They are also less reactive with sCIs and appear to be resistant to secondary chemistry, since the X + R_1_R_2_CIBr reaction, where X is any species, is more likely to produce the R_1_R_2_CBr radical (+ XI) than the R_1_R_2_CI radical (+ XBr).^11^ Thus, no additional R_1_R_2_COO is produced because the R_1_R_2_CBr + O_2_ reaction does not produce R_1_R_2_COO. Unwanted secondary reactions may lead to a chain-propagation and distort the information obtained from time-resolved kinetic measurements. In our previous studies,^11,12^ we have shown that the method utilizing R_1_R_2_CIBr precursors is preferable technique to produce sCIs, especially in experiments to measure unimolecular reaction kinetics.

Here we show that the photolysis of *c-*(CH_2_)_5_CIBr at 213 nm in the presence of O_2_ ([O_2_] ≫ [*c-*(CH_2_)_5_CI]) produces cyclohexanone oxide, *c-*(CH_2_)_5_COO, see Scheme 1. Due to its symmetric structure, cyclohexanone oxide is a single-isomer exocyclic CI with saturated C_6_-ring, see Figure 1. It is formed in the ozonolysis of methylenecyclohexane^13^ and any larger cyclic alkene with a *c-*(CH_2_)_5_C=CR_1_R_2_ moiety, such as cyclohexylideneacetone. Although not strictly atmospherically important, *c-*(CH_2_)_5_COO is the simplest model compound for bicyclic nopinone oxide (especially for its *E*-conformer, see Figure 1), which has a high yield in the tropospheric ozonolysis of β-pinene.^14−17^ Since there are no direct kinetics measurements available, the kinetics and reactivity of nopinone oxide and other cyclic sCIs are subject to large uncertainties.

**Scheme 1:**
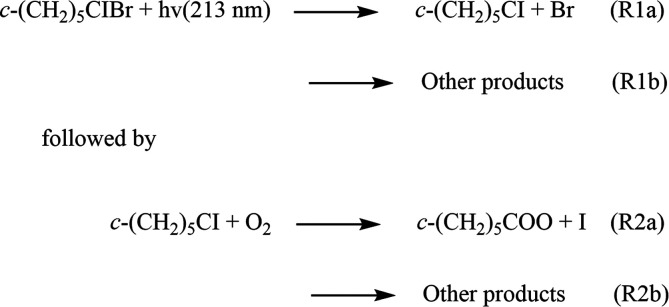
Reaction Pathway for Gas-Phase Production of *c-*(CH_2_)_5_COO As Initiated by 213 nm Photolysis of *c-*(CH_2_)_5_CIBr to Form *c-*(CH_2_)_5_CI Radicals in the Presence of O_2_

**Figure 1:**
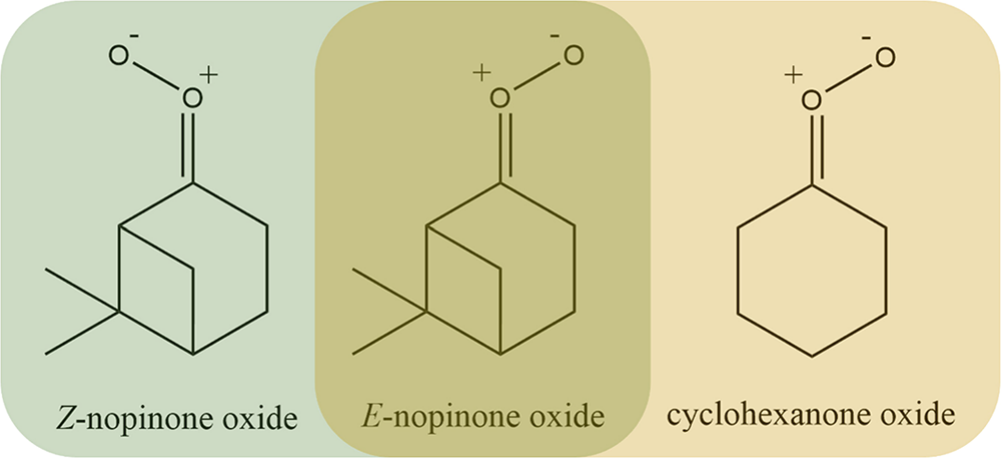
Cyclohexanone oxide and the two conformers of nopinone oxide. The *Z* and *E* refer to the orientation of the terminal oxygen atom with respect to the cyclobutyl ring.

In this work, we have performed direct kinetic measurements of the thermal unimolecular decay of *c-*(CH_2_)_5_COO, and also its bimolecular reaction with trifluoroacetic acid (TFA), CF_3_C(O)OH. We have performed the unimolecular-decay measurements over wide temperature and pressure ranges. The current results are compared with theoretical predictions of the 1,4-H-atom-shift kinetics of *E*-nopinone oxide obtained from the open literature. The unimolecular decay of *E*-nopinone oxide proceeds primarily by a 1,4-H-atom-shift reaction to form a vinyl hydroperoxide (VHP) intermediate, which quickly decomposes to form a OH radical and a cyclic, resonantly stabilized vinoxy radical.^6,15,18^ We expect *c-*(CH_2_)_5_COO to decay by the same mechanism. The bimolecular rate coefficient of the *c-*(CH_2_)_5_COO + TFA reaction is measured at 253 K and 10 Torr, and it is found to be very large. TFA is a persistent and mobile pollutant in the Earth atmosphere. It is formed mainly by oxidation of anthropogenically produced hydrofluorocarbons (HFCs), hydrochlorofluorocarbons (HCFCs) and hydrofluoro-olefins (HFOs).^19,20^ TFA reacts only slowly with OH radicals and does not photolyze easily.^21−23^ TFA, like other organic acids, reacts extremely fast with sCIs (*k*(sCI + acid) > 1 × 10^–10^ cm^3^ molecule^–1^ s^–1^).^11,24−26^

The unimolecular-decay rate coefficient (*k*_uni_) of *c-*(CH_2_)_5_COO was measured in the temperature range 213–296 K and the pressure range 7–50 Torr. Figure 2 shows typical decay traces of *c-*(CH_2_)_5_COO obtained at various initial concentrations (peak absorbances) of *c-*(CH_2_)_5_COO at 296 K and 10 Torr. The initial [*c-*(CH_2_)_5_COO]_0_ was varied by adjusting the [*c-*(CH_2_)_5_CIBr]_0_. The initial [*c-*(CH_2_)_5_COO]_0_ was estimated to be ≤1 × 10^11^ molecule cm^–3^ in all measurements (see more details in the Supporting Information). The decay of the *c-*(CH_2_)_5_COO absorption signal mainly contains contributions from the unimolecular reaction and the self-reaction, which makes the observed *c-*(CH_2_)_5_COO decay rate depend on [*c-*(CH_2_)_5_COO]_0_. The diffusive loss of *c-*(CH_2_)_5_COO and its possible reactions with other reactive species also contribute to the decay rate, but these contributions are small. The experimental decay traces of *c-*(CH_2_)_5_COO were modeled with a simplified rate equation^12,27^ (Equation S5) and fitted using Equation 1 (see Experimental Methods). The entire experimental trace signal has two distinct components: a fast decay followed by a much slower decay. The fast decay component originates from the absorption of *c-*(CH_2_)_5_COO, while the slow (background) decay corresponds to an absorbance caused by nonreactive specie(s) formed in the photolysis and/or by the unimolecular decay of *c-*(CH_2_)_5_COO. This slow decay component was observed especially in the kinetic measurements close to room temperature using high [*c-*(CH_2_)_5_COO]_0_ (see Figure 2 and Figure S8). The first term in Equation 1 represents the fast decay of *c-*(CH_2_)_5_COO, while the second term describes the slow decay of nonreactive species that are formed at the same rate (*k*_sCI_) as *c-*(CH_2_)_5_COO decays. Furthermore, Equation S7 represents an alternative fitting model to analyze the measured transient absorption signal of *c-*(CH_2_)_5_COO, which corresponds to a situation where the nonreactive species are assumed to be formed at time *t* = 0 s (i.e., in the photolysis). Both fitting models returned essentially the same values for *k*_sCI_ (differences were smaller than 0.05%), indicating that the current results are not sensitive to how the slow-decaying component was modeled in the fits (see the Supporting Information for more details).

**Figure 2:**
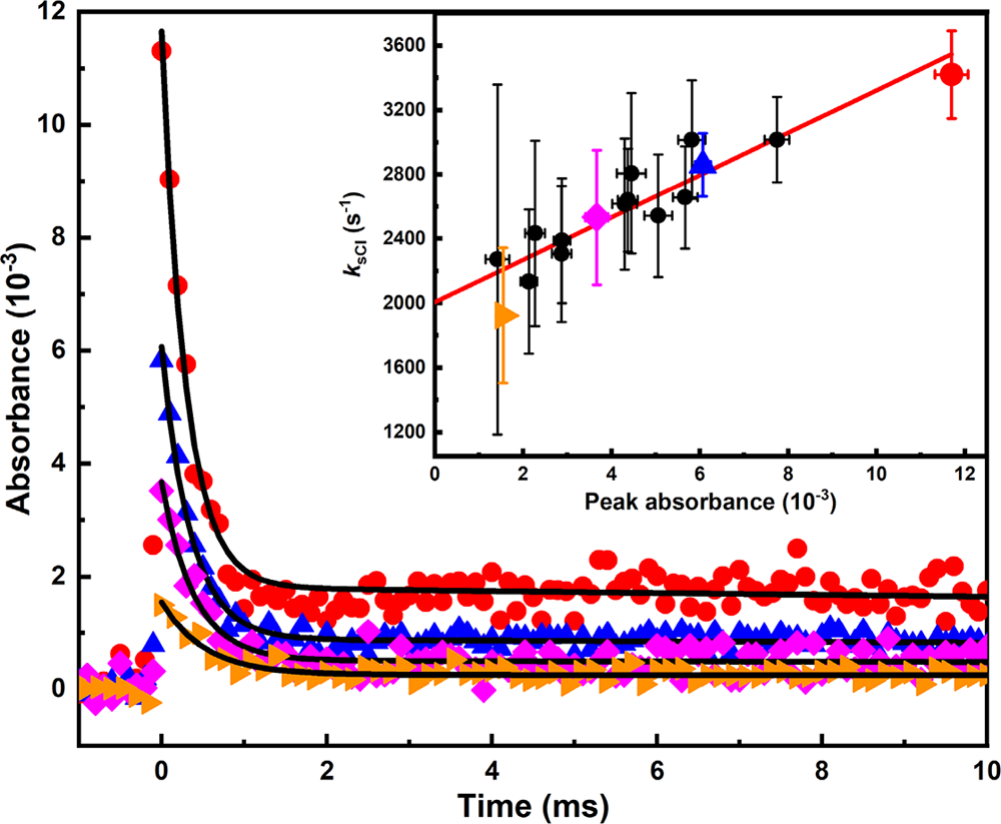
Decay traces of *c-*(CH_2_)_5_COO with 4 different initial [*c-*(CH_2_)_5_COO]_0_ at 296 K and 10 Torr. The *c-*(CH_2_)_5_COO traces were probed at 340 nm with a time resolution of 100 μs. Black curves are the fits (eq 1) to the trace. The inset shows the obtained rate coefficients (*k*_sCI_) from the fits to all the measured data as a function of peak absorbance of *c-*(CH_2_)_5_COO. Accordingly, the colored symbols in the inset represent the measurements that correspond to the shown traces. The red line is an unweighted linear least-squares fit to the data. The statistical uncertainties shown are 2σ.

A linear relationship of *k*_sCI_ with respect to [*c-*(CH_2_)_5_COO]_0_ is clearly observed (see the inset of Figure 2), indicating that the reactive species, including *c-*(CH_2_)_5_COO, are formed at concentrations proportional to [*c-*(CH_2_)_5_CIBr]_0_ in the photolysis (see more details in the Supporting Information). Extrapolating the *k*_sCI_ to zero peak absorbance ([*c-*(CH_2_)_5_COO] → 0) corresponds to the situation in which all the radical–radical reactions, such as the self-reaction, have been suppressed. Hence, the first-order decay rate coefficient of *c-*(CH_2_)_5_COO can be determined from the intercept (*k*_ic_) of the linear-least-squares fit to the obtained kinetic data. The *k*_ic_ so obtained includes the unimolecular-decay rate coefficient *k*_uni_(*c-*(CH_2_)_5_COO), and the diffusive loss rate coefficient *k*_loss_, which describes the diffusion of *c-*(CH_2_)_5_COO out of the measurement volume. The *k*_loss_ value of the current system was determined by measuring the diffusion loss of CH_2_OO under the same experimental conditions and then comparing the diffusivities of CH_2_OO and *c-*(CH_2_)_5_COO with the literature values of formic acid (HCOOH) and 4-methylpentanoic acid, C_6_H_12_O_2_ (see the Supporting Information for more details).^28^

Figure 3 shows an Arrhenius plot of the *k*_uni_(*c-*(CH_2_)_5_COO) values measured at a total density of 0.33 × 10^18^ molecule cm^–3^ (∼10 Torr). The current measurements indicate that the reaction is already at the high-pressure limit at 10 Torr. The conditions and results of our measurements are tabulated in Table 1. None of the several ozonolysis studies of β-pinene report a relative unimolecular-decay rate coefficient for nopinone oxide.^14,16,17^ Neither are there such studies for *c-*(CH_2_)_5_COO. Vereecken et al.^6^ and Nguyen et al.^15^ have computed the 1,4-H-atom-shift rate coefficient for *E*-nopinone and found it to be several orders of magnitude larger than that of the dioxirane pathway. Nguyen et al.^15^ calculated *k*_uni_(*E*-nopinone oxide) = 50 s^–1^ for the 1,4-H-atom shift reaction at room temperature and atmospheric pressure.

**Figure 3:**
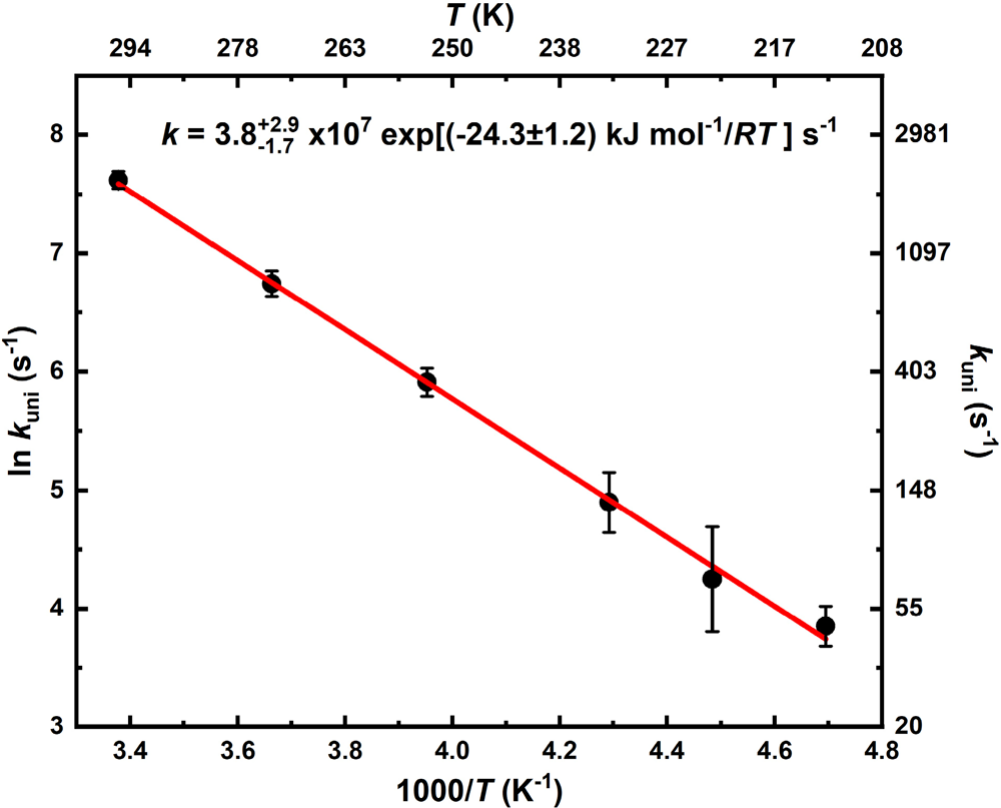
An Arrhenius plot of the unimolecular-decay reaction rate coefficients of *c-*(CH_2_)_5_COO measured in this work at the total density of 0.33 × 10^18^ molecule cm^–3^. The solid red line is an unweighted linear least-squares fit to the data, giving an Arrhenius expression of *k* = 3.8_–1.7_^+2.9^ × 10^7^ exp[(−24.3 ± 1.2) kJ mol^–1^/*RT*] s^–1^, with 2σ standard fitting uncertainties.

**Table 1:** Experimental Conditions and Unimolecular Reaction Rate Coefficients of *c-*(CH_2_)_5_COO Determined from the UV-Absorption Experiments

*T* (K)	[N_2_] (×10^18^ molecule cm^–3^)	*p*^a^ (Torr)	*k*_ic_ (s^–1^)	*k*_loss_ (s^–1^)	*k*_uni_ (s^–1^)
296	0.33	10	2006 ± 147	8	1998
296	1.60	50	1850 ± 642	4	1846
273	0.33	9.2	855 ± 91	8	847
273	1.60	46	1073 ± 217	4	1069
253	0.33	8.5	377 ± 44	8	369
253	1.60	42.6	385 ± 270	4	381
233	0.33	7.9	142 ± 34	8	134
223	0.33	7.5	78 ± 31	8	70
213	0.33	7.2	55 ± 8	8	47

a The fixed O_2_ concentration was ∼4 × 10^16^ molecule cm^–3^. *k*_ic_ is the intercept of the linear least-squares fit to the kinetic data (*k*_sCI_) measured as a function of [*c-*(CH_2_)_5_COO], with 2σ statistical fitting uncertainties. *k*_uni_ are derived as *k*_uni_ = *k*_ic_ – *k*_loss_, where *k*_loss_ is 0.39 × *k*_loss_(CH_2_OO) at a given temperature and total density (see more details in the Supporting Information).

Later, Vereecken et al.^6^ calculated a slightly larger rate coefficient for this reaction at room temperature, *k*_uni_(*E*-nopinone oxide) = 375 s^–1^. Given that the rate coefficient for this reaction is sensitive to not only the height of the barrier, but also its shape due to the massive tunnelling effect, differences in computational methodologies can easily explain the difference between the computed rate coefficients. According to the theoretical calculations, the *Z-*nopinone oxide cannot undergo the hydroperoxide channel due to the ring strain and isomerizes to a dioxirane with a slow rate of ∼1 s^–1^.^6,15^

To compare the 1,4-H-atom-shift kinetics of cyclohexanone oxide and *E-*nopinone oxide, we performed density functional theory (DFT) calculations at the MN15/Def2TZVP^29,30^ level of theory for both systems to compute activation Gibbs energies for the 1,4-H-atom-shift transition states. The computed activation Gibbs energies are about 5 kJ mol^–1^ lower for the cyclohexanone oxide system. Because of the massive tunnelling effect, the activation energy obtained from the Arrhenius fit is about a factor of 2 smaller than the zero-point-energy corrected 1,4-H-shift barrier heights. By applying the thermodynamic formulation of transition state theory (TST), we estimate that the cyclohexanone oxide system has a 5–6 times larger 1,4-H-atom-shift rate coefficient than the *E-*nopinone oxide system. This simple estimate of the reactivity difference compares well with the experimental results obtained in the current study and the room-temperature computation by Vereecken et al.^6^ At 296 K and close to the high-pressure limit, we measured *k*_uni_(*c-*(CH_2_)_5_COO) = 1998 ± 147 s^–1^ for cyclohexanone oxide, whereas Vereecken et al.^6^ obtained 375 s^–1^ for *E*-nopinone oxide. The DFT/TST calculations are explained in more detail in the Supporting Information.

Because the unimolecular-decay of *c-*(CH_2_)_5_COO is so fast already at room temperature, the bimolecular *c-*(CH_2_)_5_COO + TFA rate coefficient was determined at 253 K and 10 Torr. At 253 K, the unimolecular loss of *c-*(CH_2_)_5_COO is sufficiently slow that bimolecular rate coefficients can measured with the time-resolved broadband cavity-enhanced absorption spectrometer apparatus. The upper left corner of Figure 4 shows the transient traces of *c-*(CH_2_)_5_COO in the absence and presence of TFA at 253 K and 10 Torr. All the *c-*(CH_2_)_5_COO traces in the bimolecular study were fitted using Equation 1. In the absence of added TFA, the *c-*(CH_2_)_5_COO signal follows a first-order decay loss, *k*_loss_ (s^–1^), which is mainly due to the unimolecular decay of *c*-(CH_2_)_5_COO, and to some small extent to due to its self-reaction (low [*c-*(CH_2_)_5_COO]_0_ was used in these experiments) and diffusion out of the measurement volume. By adding a known [TFA], the decay of *c-*(CH_2_)_5_COO became faster. All the measurements were performed under pseudo-first-order conditions, i.e. [*c-*(CH_2_)_5_COO] ≪ [CF_3_C(O)OH] (see the Supporting Information for details on the determination of [CF_3_C(O)OH]).

**Figure 4:**
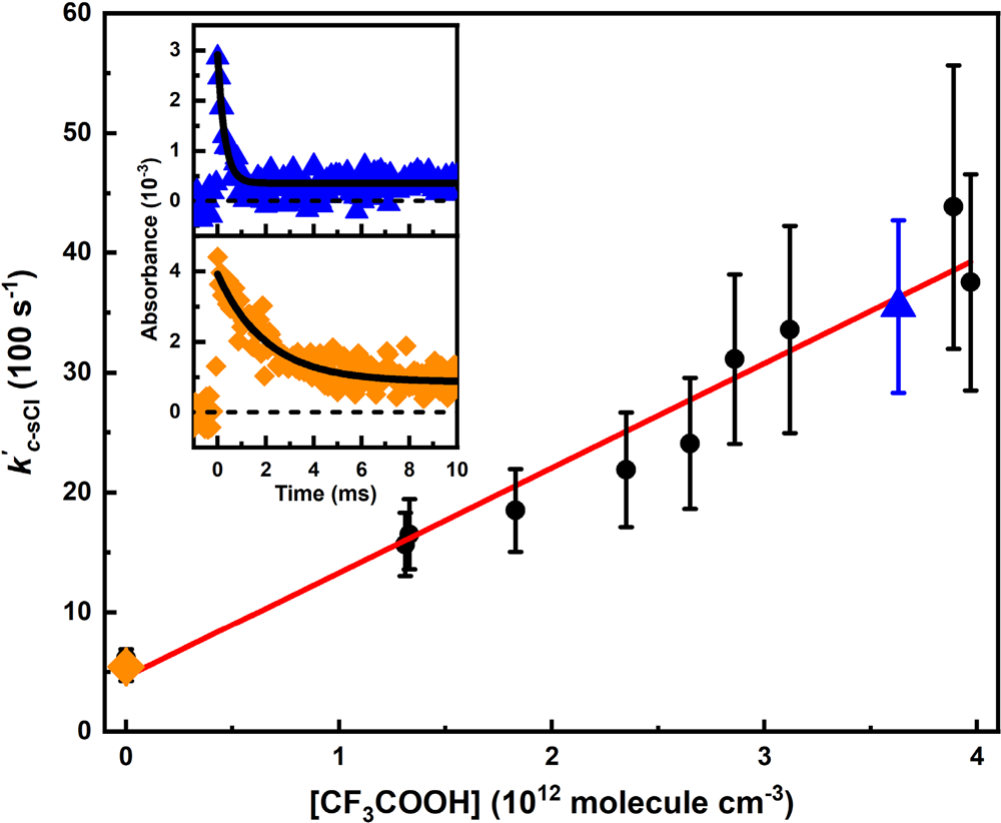
Determination of the bimolecular rate coefficient of the *c-*(CH_2_)_5_COO + CF_3_C(O)OH reaction from the plot of pseudo-first-order decay rate coefficients (*k*′_*c*-sCI_) versus [CF_3_C(O)OH] at 253 K and 10 Torr. The [*c-*(CH_2_)_5_COO] traces in the absence (orange diamonds) and presence (blue triangles) of CF_3_C(O)OH are shown in the upper left corner. Accordingly, the colored symbols in the figure represent the measurements that correspond to the shown traces. The *c-*(CH_2_)_5_COO traces were probed at 340 nm with a time resolution of 67 μs. The red line is an unweighted linear least-squares fit to the data. The statistical uncertainties shown are 2σ.

In Figure 4, the obtained pseudo-first-order decay rate coefficients of *c-*(CH_2_)_5_COO, *k*′_*c*-sCI_, are shown as function of [CF_3_C(O)OH]. The complete results and the experimental conditions of the measurements are shown in Table S3. The bimolecular rate coefficient *k*(*c-*(CH_2_)_5_COO + CF_3_C(O)OH) is obtained from the slope of the equation *k*′_*c*-sCI_ = *k*_loss_ + *k*(*c-*(CH_2_)_5_COO + CF_3_C(O)OH) × [CF_3_C(O)OH] fitted to the data, giving (8.7 ± 1.0) × 10^–10^ cm^3^ molecule^–1^ s^–1^. This result agrees with several previous determinations, where very rapid reactions of sCIs with halogenated organic acids have been measured.^24,25^ The kinetics of *c-*(CH_2_)_5_COO + TFA reaction measured in this study is slightly faster than the previously reported CH_2_OO + TFA and (CH_3_)_2_COO + TFA reactions with the rate coefficients of (3.76 ± 0.10) × 10^–10^ cm^3^ molecule^–1^ s^–1^ at 256 K and 10 Torr, and (6.71 ± 0.26) × 10^–10^ cm^3^ molecule^–1^ s^–1^ at 259 K and 10 Torr, respectively.^24^

In summary, this work introduces a new photolytic precursor of the cyclic stabilized Criegee intermediate, 1-bromo-1-iodocyclohexane, which photolysis at 213 nm in the presence of O_2_ produces cyclohexanone oxide, *c-*(CH_2_)_5_COO. This new photolytic method is a significant step toward direct kinetic studies of cyclic sCIs reactions. Using this method, we have performed direct kinetic measurements of the thermal unimolecular reaction of *c-*(CH_2_)_5_COO and its bimolecular reaction with trifluoroacetic acid.

## Experimental Methods

All the kinetic experiments were performed using a time-resolved broadband cavity-enhanced absorption spectrometer apparatus that is schematically shown in Figure S1 and has been described previously.^11,12,31^ Cyclohexanone oxide was produced homogeneously along the reactor by the pulse-photolysis of *c-*(CH_2_)_5_CIBr at 213 nm in the presence of O_2_ ([O_2_] ≫ [*c-*(CH_2_)_5_CI]), see Scheme 1. The novel precursor compound *c-*(CH_2_)_5_CIBr was synthesized as part of this work (see the Supporting Information for more details). The premixed gas mixture flowing through the temperature-controlled reactor contained the radical precursor *c-*(CH_2_)_5_CIBr, O_2_, and TFA (for the bimolecular reaction measurements only) diluted in nitrogen carrier gas. All the kinetic absorption traces were measured at 340 nm with a time resolution of 50–150 μs (typically 100 μs). The measured absorption spectrum of *c-*(CH_2_)_5_COO is shown in Figure S6. The low cavity transmission below 330 nm prevented accurate measurement of the spectrum at short wavelengths (see the Supporting Information for more details). The following equation was fitted to the transient absorption signal of *c-*(CH_2_)_5_COO,
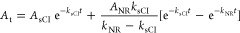
1where *A*_t_ is the measured absorbance at time *t*, *k*_sCI_ is the first-order decay rate coefficient of *c-*(CH_2_)_5_COO to be obtained, *A*_sCI_ is the initial absorbance of *c-*(CH_2_)_5_COO (at time *t* = 0), *k*_NR_ is the obtained first-order decay rate coefficient of nonreactive species, and *A*_NR_ is the maximum absorbance of nonreactive species. The statistical fitting uncertainties shown in this study are 2σ. This includes uncertainties of all the measured exponential decays (*k*_sCI_ and *k*′_c-sCI_) and linear least-squares fits. The estimated overall uncertainty in the reported unimolecular rate-coefficient values is ±20%. More experimental details can be found in the Supporting Information of this letter.

## Supporting Information Available

Additional experimental details, spectrum of *c-*(CH_2_)_5_COO, synthesis of the precursor, 400 MHz ^1^H NMR spectrum of the precursor, 100 MHz ^13^C NMR spectrum of the precursor, HRMS measurements of the precursor, attenuated total reflectance (ATR) spectrum of the precursor, details of the DFT and TST calculations, and details of the unimolecular and bimolecular kinetic measurements of *c-*(CH_2_)_5_COO (PDF)
Transparent Peer Review report available (PDF)
