# Supplementary material for: Direct Kinetic Measurements of a Cyclic Criegee Intermediate; Unimolecular Decomposition of c-(CH2)5COO
Source: J Phys Chem Lett. 2024 May 10;15(20):5331–6. doi: 10.1021/acs.jpclett.4c00554 (PMC11389976; doi:10.1021/acs.jpclett.4c00554)
Supplement: Supplementary file 1 — jz4c00554_si_001.pdf [file jz4c00554_si_001.pdf]

Supporting Information

for

Direct Kinetic Measurements of a Cyclic Criegee Intermediate; Unimolecular Decomposition of *c*-$(CH_2)_5COO$

*Jari Peltola, Petri Heinonen, and Arkke Eskola**

Department of Chemistry, University of Helsinki, P.O. Box 55 (A.I. Virtasen aukio 1), FI-00014, Helsinki, Finland

Corresponding Author

*arkke.eskola@helsinki.fi

Table of Contents

Table of Contents S2
Experiments S2
Spectrum of *c*-$(CH_2)_5COO$ S7
Unimolecular reaction of *c*-$(CH_2)_5COO$ S7
DFT and TST calculations S11
Bimolecular reaction of *c*-$(CH_2)_5COO$ with $CF_3C(O)OH$ S12
References S13

Experiments

A schematic figure of the time-resolved, broadband, cavity-enhanced absorption spectroscopy (TR-BB-CEAS) apparatus utilizing UV absorption to probe *c*-$(CH_2)_5COO$ is shown in Figure S1 and has been described in detail in our previous publications.[1-3] Figure S2a shows the cavity transmission as a function of wavelength, while Fig. S2b shows the effective optical path length (OPL) of the TR-BB-CEAS cavity as a function of wavelength. The OPL was determined using a static (5.07 ± 0.10) ppm NO_2 in N_2 -sample in the reactor at 20 Torr and 296 K. The observed absorbance ($A_{\mathrm{NO_2},\lambda}$) between wavelengths of 300 nm and 450 nm is

$$A_{\mathrm{NO_2},\lambda} = \ln\frac{I_{0,\lambda}}{I_\lambda} = \sigma_{\mathrm{NO_2},\lambda} \times [\mathrm{NO_2}] \times OPL_\lambda \qquad \text{(S1)}$$

where $I_{0,\lambda}$ is the light intensity at wavelength λ in the absence of NO_2, I_λ is the light intensity at wavelength λ in the presence of the static NO_2 sample, $\sigma_{\mathrm{NO_2},\lambda}$ is the NO_2 absorption cross-section at wavelength λ, $[NO_2]$ is the NO_2 concentration, and the OPL_λ is the effective optical path length at wavelength λ. Using Equation S1, the effective OPL is

$$OPL = \frac{A_{\mathrm{NO_2},\lambda}}{\sigma_{\mathrm{NO_2},\lambda} \times [\mathrm{NO_2}]} \qquad \text{(S2)}$$

The average effective OPL at 340 nm (with the wavelength resolution of 4 nm) is about 75 m. The initial concentration of *c*-$(CH_2)_5COO$ is estimated from the observed initial absorbance ($A_{0,c\text{-}(\mathrm{CH_2})_5\mathrm{COO}}$) at 340 nm as

$$[c\text{-}(\mathrm{CH_2})_5\mathrm{COO}]_0 = \frac{A_{0,c\text{-}(\mathrm{CH_2})_5\mathrm{COO}}}{\sigma_{\mathrm{Criegee}} \times OPL} \qquad \text{(S3)}$$

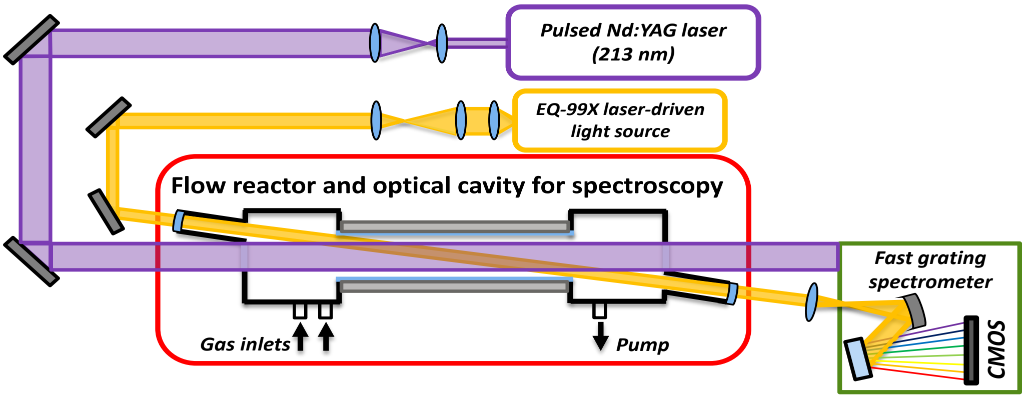

Figure S1. Schematic figure of the time-resolved, broadband, cavity-enhanced absorption spectrometer. The sCI is produced along a flow tube reactor by a single-pass photolysis laser pulse at 213 nm. The sCI is probed by overlapping incoherent laser-driven broadband light source. The sensitivity of the detection is enhanced using an optical cavity formed by two highly reflecting concave mirrors between 300 and 450 nm. The time-dependent broadband absorption spectrum of [sCI] is measured by a grating spectrometer combined with a fast CMOS line array camera.

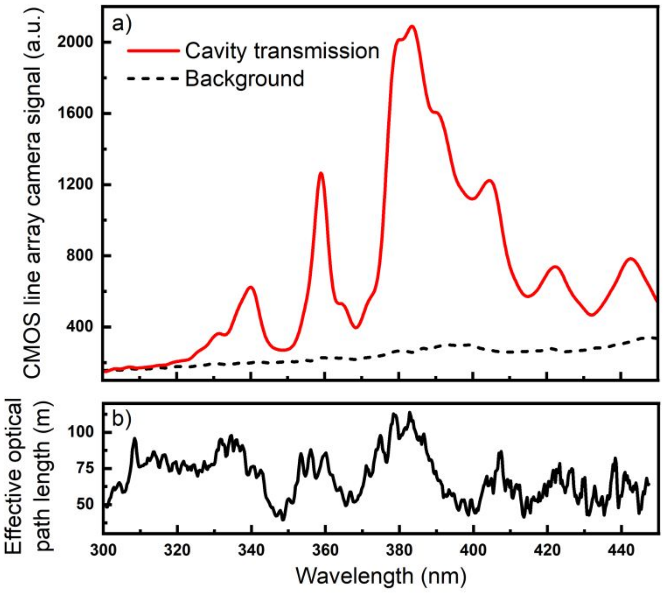

Figure S2. a) The cavity transmission signal. b) The effective optical path length of the TR-BB-CEAS apparatus as a function of wavelength.

Note that the absorption cross-section of *c*-$(CH_2)_5COO$ (or *c*-$(CH_2)_5CIBr$) has not been measured and it is unknown at 340 nm. Here, the initial *c*-$(CH_2)_5COO$ concentration in the measurements has been estimated assuming that its absorption cross-section ($\sigma_{Criegee}$) at 340 nm is of the same order of magnitude as that of acetone oxide[4] ($(CH_3)_2COO$) and propionaldehyde oxide[5] (CH_3CH_2CHOO), which are about 2×10^{-17} cm^2 $molecule^{-1}$ at 340 nm. Of all measurements, the highest peak absorbance signal (~0.012, Fig. 2) gives the highest initial concentration of $\sim 8.0 \times 10^{10}$ molecules cm^{-3} for *c*-$(CH_2)_5COO$. Thus, the initial [*c*-$(CH_2)_5COO]_0$ was estimated to be ≤ 1 × 10^{11} molecule cm^{-3} in all measurements.

The method to cool the flow tube reactor is described in our previous publication.[2, 3] Two temperature-controlled custom-made aluminum blocks were placed around the stainless-steel reactor tube, which inner wall was coated with halocarbon wax. The aluminum blocks were fitted with copper tubes connected to an external cooling bath circulation (Huber CC-905). Ethanol was employed as a heat transfer fluid for the cooling. The gases were pre-cooled close to the setpoint temperature before entering the reactor. The complete axial temperature profile within the overlap volume of the probe and the photolysis beams were measured separately for all experimental conditions (temperature, pressure, and flow rate) used in this work. The observed temperature 2σ uncertainty in the measurement range 213–296 K was ≤ ±1.5 K.

Synthesis of the 1-bromo-1-iodocyclohexane precursor: 1-Bromocyclohexene[6] (8.7 g, 54 mmol) was dissolved in trifluoroacetic acid (25 ml) and concentrated aqueous hydroiodic acid (57 wt%, 12.3 ml, 92 mmol) was added. The reaction was monitored by ^1H NMR and stopped after 6,5 h. The reaction mixture was diluted with pentane, washed 3 times with water, then with 10 % aq $Na_2S_2O_3$ and finally with water 3 more times. The organic phase was dried over $MgSO_4$ and evaporated. The distillation twice at reduced pressure (58 °C, 0.8 mbar) gave the title compound (7.1 g, 45 %). ^1H NMR (400 MHz, $CDCl_3$) δ 2.68 – 2.62 (2H, m), 2.39 – 2.34 (2H, m), 1.76 – 1.66 (2H, m), 1.60 – 1.47 (4H, m). ^{13}C NMR (100 MHz, $CDCl_3$) δ 52.6, 44.7, 27.0, 24.5. MS (EI^+, 40 eV): *m/z* (%): 81 (100), 161 (40), 163 (40), 208 (40), 288 (0.5) 290 (0.5). HRMS (EI^+, 40 eV): *m/z* : $[M]^+$ calcd for $C_6H_{10}{}^{79}BrI$: 287.9011 found: 287.8998, $[M]^+$ calcd for $C_6H_{10}{}^{81}BrI$: 289.8990 found: 289.999, $[M–Br]^+$ calcd for $C_6H_{10}I$: 208.9827 found: 208.9827, $[M–I]^+$ calcd for $C_6H_{10}{}^{79}Br$: 160.9966 found: 160.9965, $[M–I]^+$ calcd for $C_6H_{10}{}^{81}Br$: 162.9945 found:162.9952. IR (ATR): *v* = 2933, 2855, 1444, 1331, 1261, 1242, 1116, 995, 685 cm^{-1}. According to NMR analysis, the precursor product contained a small amount (1-3 wt%) of the 1-bromocyclohexene reagent. The residual trifluoroacetic acid concentration in the product was negligible due to two vacuum distillations.

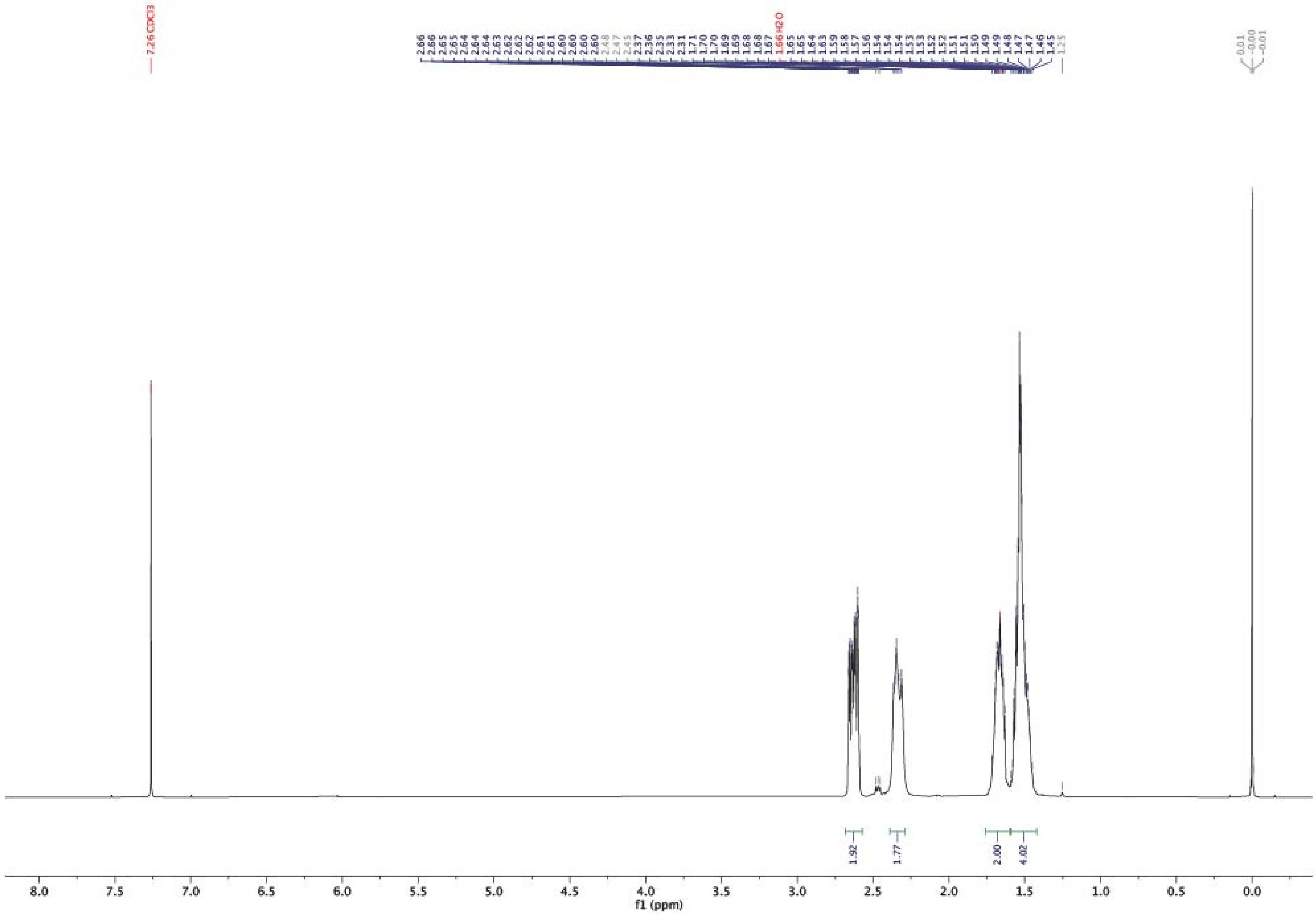

Figure S3. 400 MHz ^{1}H NMR spectrum of 1-bromo-1-iodocyclohexane in $CDCl_3$.

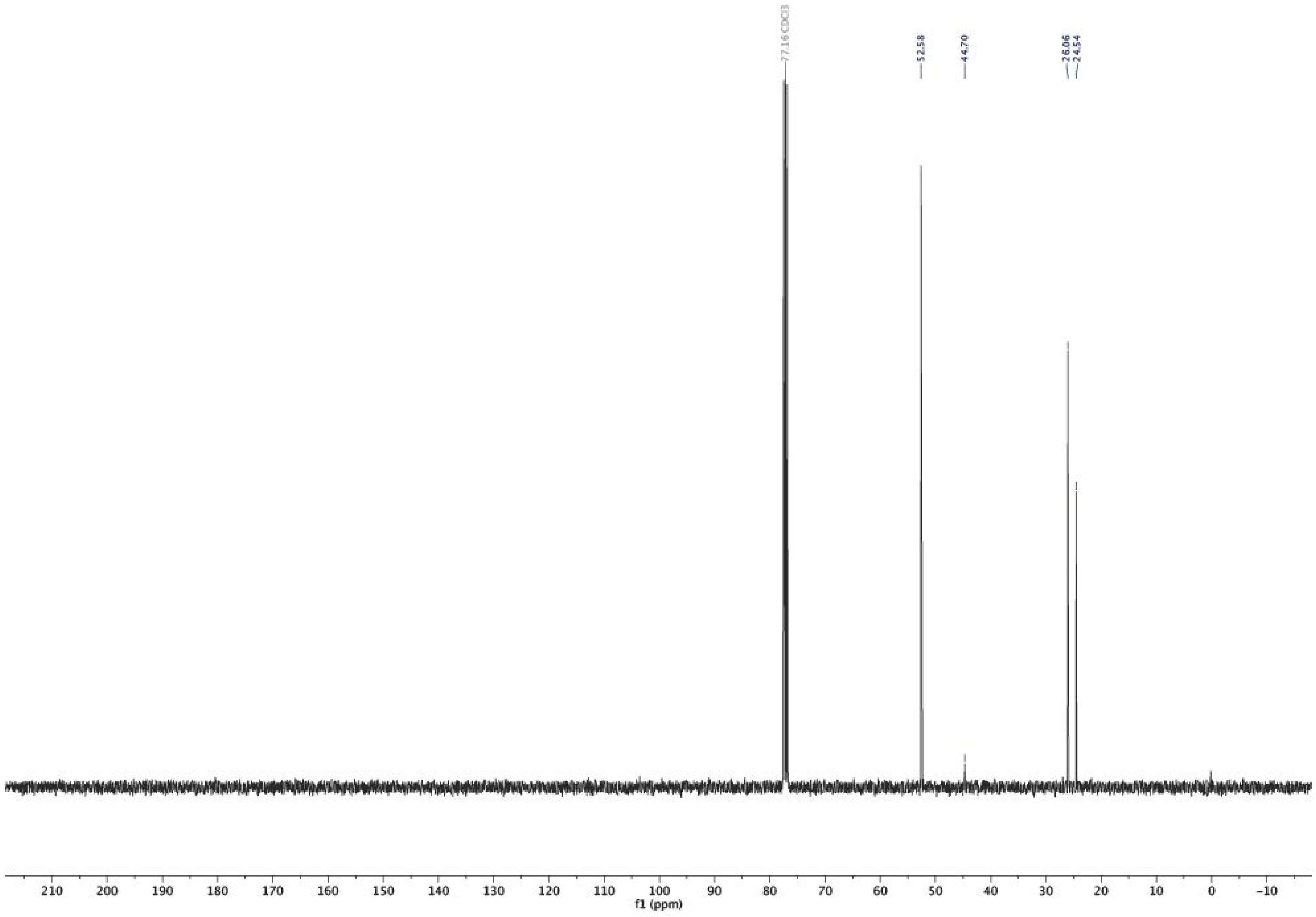

Figure S4. 100 MHz ^{13}C NMR spectrum of 1-bromo-1-iodocyclohexane in $CDCl_3$.

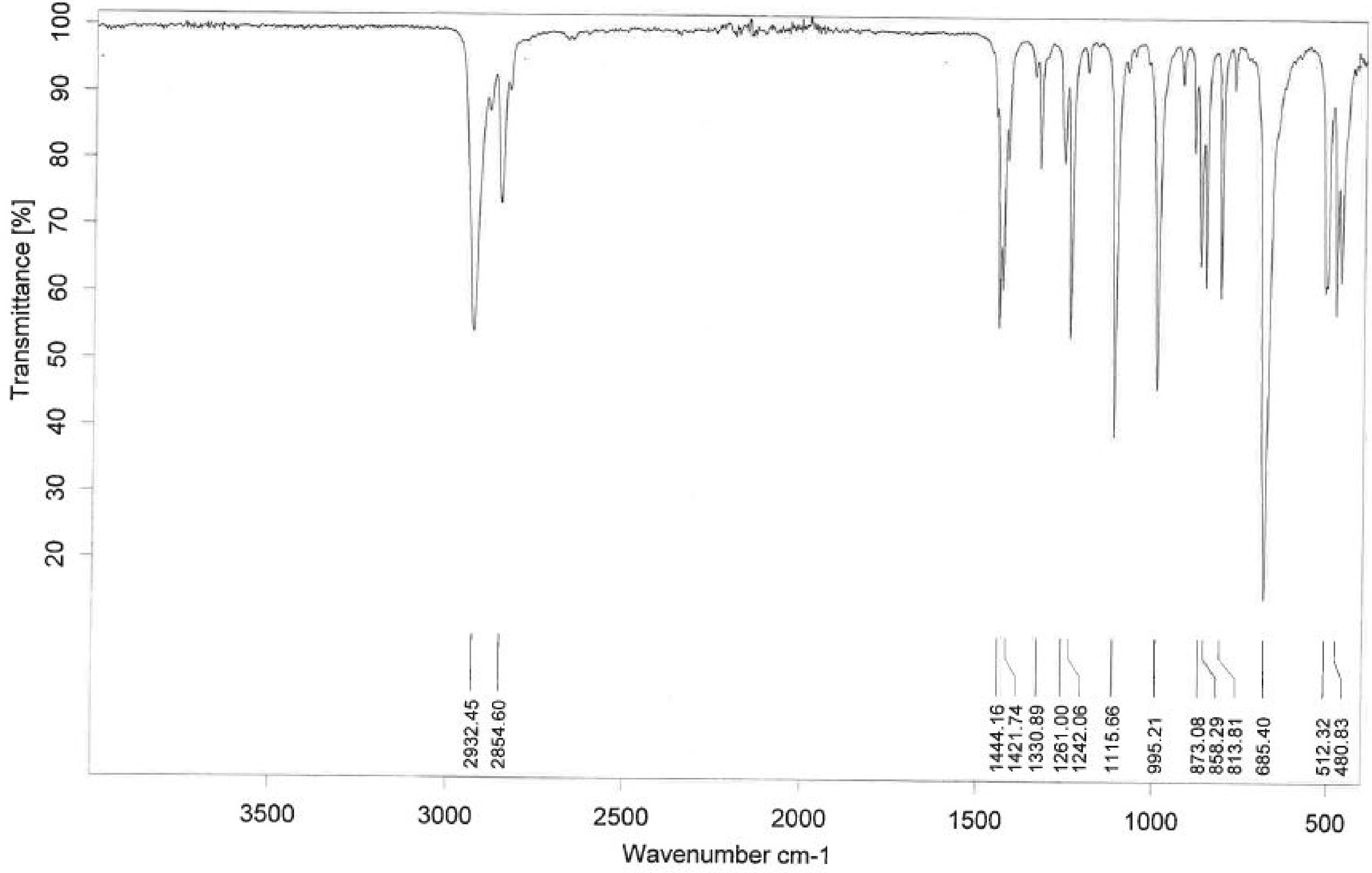

Figure S5. The attenuated total reflectance (ATR) spectrum of 1-bromo-1-iodocyclohexane in the infrared region.

Spectrum of c-(CH$_2$)$_5$COO

The absorption spectrum of c-(CH$_2$)$_5$COO measured in this work is shown with blue circles in Fig. S6 together with the fitted IO spectrum (red line). The spectrum has been measured 0.2 ms after the photolysis laser pulse with a time resolution of 100 µs and has been averaged 4000 times. In addition to the UV spectrum of c-(CH$_2$)$_5$COO, the spectrum contains other overlapping spectral features. The largest interference comes from the formation of IO, which is formed mostly by secondary chemistry. Under current experimental conditions, IO does not react with c-(CH$_2$)$_5$COO (or $CF_3C(O)OH$) and decays slowly on the experimental timescale due to diffusion out of the measurement volume. The depletion of c-(CH$_2$)$_5$ClBr does not cause a (negative) interference to the spectrum due to the low absorption (see more in the next paragraph). The IO spectrum was fitted to the measured data (together with a constant background of 0.15 cm^{-1}), from which [IO] = 3.4×10^{10} molecule cm^{-3} was determined. The estimated initial c-(CH$_2$)$_5$COO concentration in the measurement was <1.0×10^{11} molecule cm^{-3}. The low cavity transmission (see Fig. S2a) below 330 nm inhibits accurate measurement of the spectrum at short wavelengths.

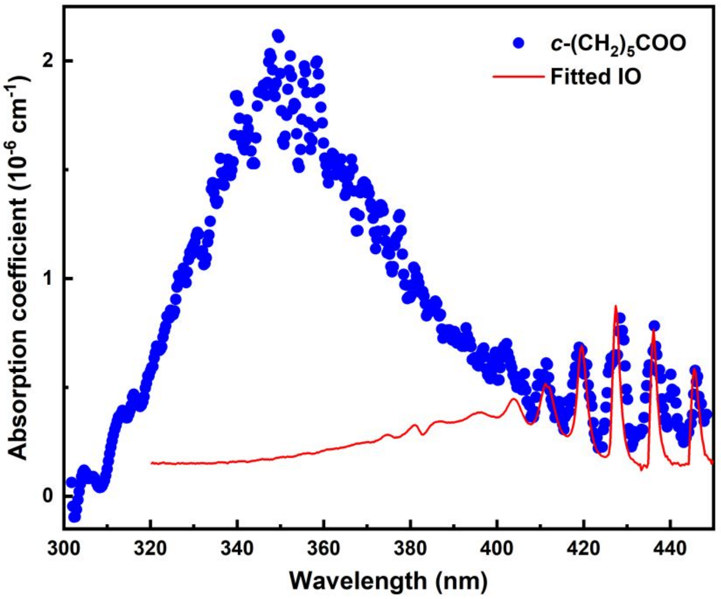

Figure S6. Absorption spectrum of c-(CH$_2$)$_5$COO measured at 253 K and 8.5 Torr. The spectrum is averaged 4000 times over t = 0–100 µs. The initial c-(CH$_2$)$_5$COO concentration was estimated to be <1.0×10^{11} molecule cm^{-3}. The O_2 concentration was 4.0×10^{16} molecule cm^{-3}.

Thus, the shape of the absorption profile is distorted, and the absorption is most likely stronger at shorter wavelengths than Figure S6 suggest. To our knowledge, there is no theoretical calculation for the absorption cross-section of c-(CH$_2$)$_5$COO. However, the current measured spectrum of c-(CH$_2$)$_5$COO resembles the spectra of other Criegee intermediates.[7, 8] Although we cannot precisely determine the spectrum at short wavelengths, its shape is evidence that the measured spectrum originates from c-(CH$_2$)$_5$COO. In particular, the very large bimolecular reaction coefficient obtained in this study for the reaction with $CF_3C(O)OH$ indicates that the compound formed in the reaction c-(CH$_2$)$_5$Cl + O_2 is c-(CH$_2$)$_5$COO.

Unimolecular reaction of c-(CH$_2$)$_5$COO

As is mentioned in the main text, the observed decay of c-(CH$_2$)$_5$COO mainly contain contributions from the thermal unimolecular reaction and self-reaction, but also a small contribution from the gas diffusion loss and to some small extend from the possible reaction with other reactive species. The rate equation to describe the loss of c-(CH$_2$)$_5$COO can be written as

$$-\frac{d[c\text{-}(\mathrm{CH_2})_5\mathrm{COO}]}{dt} = k_{\mathrm{uni}}[c\text{-}(\mathrm{CH_2})_5\mathrm{COO}] + 2k_{\mathrm{self}}[c\text{-}(\mathrm{CH_2})_5\mathrm{COO}]^2 + k_{\mathrm{bi}}[\mathrm{X}][c\text{-}(\mathrm{CH_2})_5\mathrm{COO}] + k_{\mathrm{loss}}[c\text{-}(\mathrm{CH_2})_5\mathrm{COO}]$$
$$= (k_{\mathrm{uni}} + k_{\mathrm{loss}} + 2k_{\mathrm{self}}[c\text{-}(\mathrm{CH_2})_5\mathrm{COO}] + k_{\mathrm{bi}}[\mathrm{X}])[(c\text{-}\mathrm{CH_2})_5\mathrm{COO}], \quad \text{(S4)}$$

where k_{uni} is the unimolecular reaction rate coefficient, k_{self} is the rate coefficient for the self-reaction c-(CH$_2$)$_5$COO + c-(CH$_2$)$_5$COO, [X] is the total concentration of other possible reactive species contributing to the bimolecular loss (originating, for example, from c-(CH$_2$)$_5$COO + Br and c-(CH$_2$)$_5$COO + c-(CH$_2$)$_5$ClOO reactions) with a combined rate coefficient k_{bi}, and k_{loss} is the diffusion loss rate coefficient. Although the self-reaction rate coefficient for c-(CH$_2$)$_5$COO is probably large, on the order of 10^{-10} cm^3 molecule^{-1} s^{-1}, the thermal unimolecular reaction is the main loss mechanism due to the low initial c-(CH$_2$)$_5$COO concentration ([c-(CH$_2$)$_5$COO]$_0$ < 10^{11} molecule cm^{-3}) used in the measurements. The decay traces of c-(CH$_2$)$_5$COO were fitted to an integrated first order decay expression

(Equation S6) which is derived from the simplified model (Equation S5), where the term $2k_{self}[c\text{-(CH}_2)_5\text{COO}] + k_{bi}[X]$ of Equation S4 is approximated as a constant $k_{eff}[X_{eff}]$[9]

$$-\frac{d[c\text{ - (CH}_2)_5\text{COO}]}{dt} = (k_{\text{uni}} + k_{\text{loss}} + k_{\text{eff}}[\text{X}_{\text{eff}}])[c\text{ - (CH}_2)_5\text{COO}] = k_{\text{sCI}}[c\text{ - (CH}_2)_5\text{COO}] \quad \text{(S5)}$$

The integrated first-order decay expression is

$$[c\text{ - (CH}_2)_5\text{COO}]_\text{t} = [c\text{ - (CH}_2)_5\text{COO}]_0 \times \exp(-k_{\text{sCI}}) \quad \text{(S6)}$$

where k_{SCI} is the obtained first-order decay rate coefficient, $[c\text{-(CH}_2)_5\text{COO}]_t$ is the c-$(CH_2)_5COO$ concentration at time t, and $[c\text{-(CH}_2)_5\text{COO}]_0$ is the initial c-$(CH_2)_5COO$ concentration (at time t=0). This simplified method to describe the loss of c-$(CH_2)_5COO$ and the use of single-exponential fitting method has been tested previously by Smith et al.[9] and us[2] in a similar unimolecular decompositioin study of $(CH_3)_2COO$. The single-exponential function fits reasonably well with the absorption traces, although the observed absorbance can be slightly higher than the fitted value just after the photolysis. In the main text, the inset of Figure 2 shows the linear relationship between the obtained first-order decay rate coefficients (k_{sCI}) and the $[c\text{-(CH}_2)_5\text{COO}]_0$ at 296 K and 10 Torr. The linear behavior suggests that $[X_{eff}]$ is proportional to $[c\text{-(CH}_2)_5\text{COO}]_0$. This is expected since most of the reactive species, as well as c-$(CH_2)_5COO$, are formed at small concentrations proportional to $[c\text{-(CH}_2)_5\text{ClBr}]_0$ and the laser pulse energy (assuming there are no other reactive species in the precursor mixture). Extrapolating the k_{sCI} to zero $[c\text{-(CH}_2)_5\text{COO}]_0$ removes the effect of radical – radical processes, including contributions from c-$(CH_2)_5COO$ + Br and c-$(CH_2)_5COO$ + c-$(CH_2)_5ClOO$ reactions. Hence, the unimolecular reaction rate coefficient of c-$(CH_2)_5COO$ can be determined from the intercept (k_{ic}) of the linear least squares fit to the obtained kinetic data. Although a more accurate kinetics model would give better fits to the absorption traces, the intercept values of the more accurate and simplified models would be almost the same since the reactive species are formed at small concentrations. These concentrations are proportional to $[c\text{-(CH}_2)_5\text{ClBr}]_0$ and the laser pulse energy. Smith et al.[9] have stated this previously in their study.

As is mentioned in the main text, the entire experimental trace signal has two distinct components: a fast decay and a much slower decay. The fast decay component originates from the (decaying) absorption of c-$(CH_2)_5COO$, while the much slower (background) decay corresponds to an absorbance caused by nonreactive specie(s) formed in the photolysis, in the c-$(CH_2)_5Cl$ + O_2 reaction and/or in the unimolecular reaction of c-$(CH_2)_5COO$. In the fitting using Equation 1, we assume that the nonreactive species are formed during the unimolecular reaction with the same rate (k_{sCI}) as c-$(CH_2)_5COO$ decays and absorb at the detection wavelength.

The slow decay component was observed in all kinetic measurements, but especially in the measurements using higher $[c\text{-(CH}_2)_5\text{COO}]_0$ and higher total pressure (see Fig. 2 and Fig. S7-S9). Under constant conditions (T and p), the absorption of nonreactive specie(s) increases when $[c\text{-(CH}_2)_5\text{COO}]_0$ increases but maintains its relative value with respect to c-$(CH_2)_5COO$ absorbance (see Table S1 and S3). However, the value of additional absorption relative to c-$(CH_2)_5COO$ absorbance increases with increasing pressure, which may originate from an enhanced stabilization of c-$(CH_2)_5ClOO$ radical. Correspondingly, as the yield of c-$(CH_2)_5ClOO$ radical increases at higher pressure, the yield of of c-$(CH_2)_5COO$ decreases. Depletion of c-$(CH_2)_5ClBr$, as well as other R_1R_2ClBr,[1, 2] does not cause a negative baseline shift of the measured absorption signal, like the use of $R_1R_2CI_2$ precursors,[9, 10] due to the significantly lower absorption of c-$(CH_2)_5ClBr$ at 340 nm region.

Alternatively, Equation S7 was also fitted to the experimental signals presented in Figure S8. Fittings using Equation S7 are shown in Figure S9,

$$A_\text{t} = A_{\text{sCI}}e^{-k_{\text{sCI}}t} + A_{\text{NR}}e^{-k_{\text{NR}}t} \quad \text{(S7)}$$

which corresponds to the situation where the positive slowly decaying absorption is formed at time t=0 s (in the photolysis). Table S2 shows a comparison of the results of both fittings. With a few exceptions, the obtained k_{sCI} values are identical to the accuracy of the four displayed numbers, regardless of the fitting method. Overall, the deviation was less than 0.05%. The only significant difference is the obtained lower peak absorbance (A_{sCI}) of the Criegee intermediate when using Equation S2. Using Eq. S2, the intercept (k_{IC}) of the unweighted linear least squares fit to the obtained rate coefficients (k_{sCI}) as a function of peak absorbance of c-$(CH_2)_5COO$ is 2000 ± 144 s^{-1} (see figure S10), which is essentially the same value than when using Eq. 1 (2006 ± 147 s^{-1}). This shows that regardless of the exact mechanism of positive slowly decaying baseline offset formation, the current fittings and results are reliable.

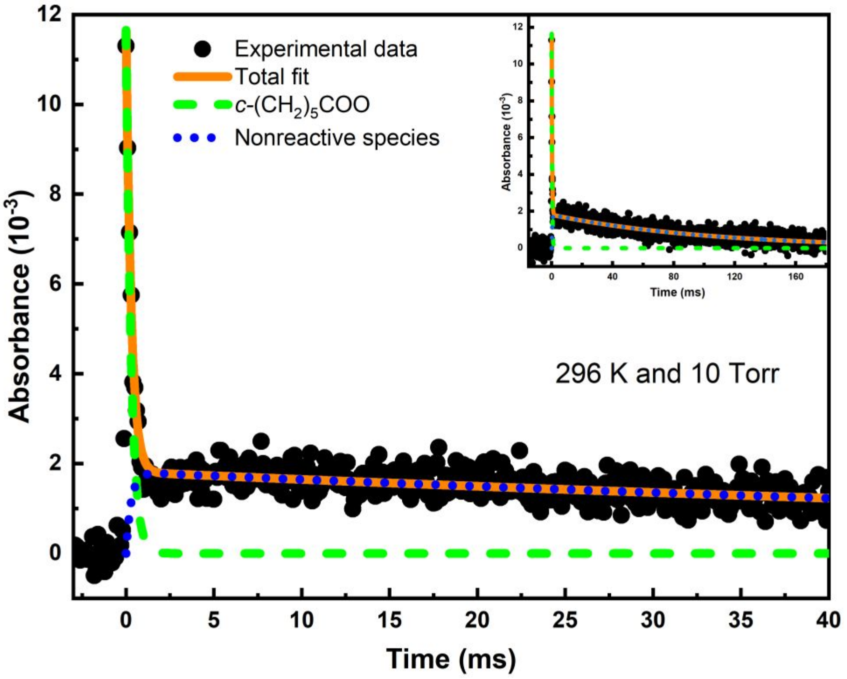

Figure S7. The transient absorption signal of *c*-$(CH_2)_5COO$ at 269 K and 10 Torr (taken from Figure 2) fitted with the Equation 1. The inset shows the entire signal. The *c*-$(CH_2)_5COO$ trace was probed at 340 nm with a time resolution of 100 μs.

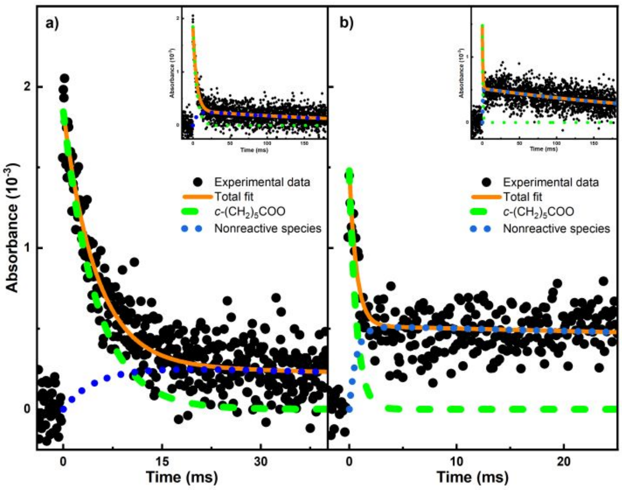

Figure S8. The transient absorption signals of c-$(CH_2)_5COO$ at a) 233 K and 7.9 Torr and at b) 273 K and 46 Torr fitted with Equation 1. The insets show the entire signal. The c-$(CH_2)_5COO$ traces were probed at 340 nm with a time resolution of 100 μs.

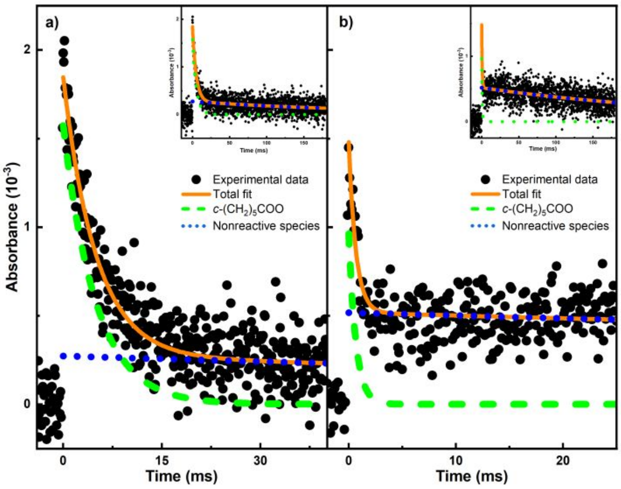

Figure S9. The transient absorption signals of *c*-$(CH_2)_5COO$ at a) 233 K and 7.9 Torr and at b) 273 K and 46 Torr from Figure S8 fitted with Equation S7. The inset shows the entire signal. The c-$(CH_2)_5COO$ traces were probed at 340 nm with a time resolution of 100 μs.

Table S1. Results of the fits to the experimental transient absorption signals presented in Fig. 2 and S7-S9.

			Using Equation 1					Using Equation S7						
***T*(K)**	Total number density ($\times 10^{18}$ molecule cm^{-3})	*p* (Torr)	k_{sCI} (s^{-1})	k_{NR} (s^{-1})	A_{sCI} ($\times 10^{-3}$)	A_{NR} ($\times 10^{-3}$)	A_{NR}/A_{sCI} (%)	k_{sCI} (s^{-1})	k_{NR} (s^{-1})	A_{sCI} ($\times 10^{-3}$)	A_{NR} ($\times 10^{-3}$)	A_{NR}/A_{sCI} (%)	$k_{sCI(Eq. 1)}$ / $k_{sCI(Eq. S7)}$ (%)	Number of averages
**296**	0.33	10	3420 ± 273	10 ± 0.3	11.65 ± 0.46	1.81 ± 0.04	15.5	3419 ± 273	10 ± 0.3	9.84 ± 0.46	1.82 ± 0.04	18.5	0.03	2237
	0.33	10	3017 ± 265	10 ± 0.4	7.75 ± 0.35	1.15 ± 0.03	14.8	3017 ± 265	10 ± 0.4	6.60 ± 0.35	1.15 ± 0.03	17.4	<0.01	3723
	0.33	10	2858 ± 198	7 ± 0.3	6.07 ± 0.22	0.88 ± 0.02	14.5	2858 ± 198	7 ± 0.3	5.18 ± 0.22	0.88 ± 0.02	17.0	<0.03	8784
	0.33	10	3016 ± 371	6 ± 0.5	5.82 ± 0.38	0.70 ± 0.03	12.0	3016 ± 371	6 ± 0.5	5.12 ± 0.38	0.70 ± 0.03	13.7	<0.01	2393
	0.33	10	2657 ± 319	7 ± 0.5	5.67 ± 0.36	0.83 ± 0.03	14.6	2656 ± 319	7 ± 0.5	4.84 ± 0.36	0.83 ± 0.03	17.1	0.04	2381
	0.33	10	2543 ± 384	8 ± 0.7	5.06 ± 0.40	0.72 ± 0.03	14.2	2543 ± 384	8 ± 0.6	4.34 ± 0.40	0.72 ± 0.02	16.6	<0.01	2442
	0.33	10	2807 ± 501	8 ± 0.7	4.45 ± 0.41	0.67 ± 0.03	15.1	2807 ± 501	8 ± 0.7	3.78 ± 0.41	0.67 ± 0.03	17.7	<0.01	1880
	0.33	10	2639 ± 323	7 ± 0.5	4.37 ± 0.28	0.58 ± 0.02	13.3	2640 ± 323	7 ± 0.5	3.79 ± 0.28	0.58 ± 0.02	15.3	0.04	5172
	0.33	10	2616 ± 410	7 ± 0.6	4.30 ± 0.35	0.61 ± 0.03	14.2	2616 ± 410	7 ± 0.6	3.68 ± 0.35	0.61 ± 0.03	16.6	<0.01	2566
	0.33	10	2532 ± 421	5 ± 0.6	3.67 ± 0.32	0.51 ± 0.01	13.9	2533 ± 422	5 ± 0.6	3.17 ± 0.32	0.51 ± 0.01	16.1	0.04	3823
	0.33	10	2385 ± 391	7 ± 0.4	2.88 ± 0.23	0.49 ± 0.02	17.0	2385 ± 391	7 ± 0.4	2.38 ± 0.23	0.49 ± 0.02	20.6	<0.01	4097
	0.33	10	2306 ± 424	6 ± 0.7	2.87 ± 0.28	0.39 ± 0.02	13.6	2306 ± 424	6 ± 0.7	2.48 ± 0.28	0.39 ± 0.02	15.7	<0.01	3286
	0.33	10	2434 ± 580	2 ± 0.5	2.27 ± 0.28	0.38 ± 0.02	16.7	2434 ± 580	2 ± 0.5	1.89 ± 0.28	0.38 ± 0.02	20.1	<0.01	5168
	0.33	10	2135 ± 450	7 ± 0.9	2.13 ± 0.24	0.29 ± 0.02	13.6	2135 ± 450	7 ± 0.9	1.83 ± 0.24	0.29 ± 0.02	15.8	<0.01	5805
	0.33	10	1923 ± 423	4 ± 0.4	1.55 ± 0.17	0.26 ± 0.01	16.8	1923 ± 423	4 ± 0.4	1.29 ± 0.17	0.26 ± 0.01	20.2	<0.01	6228
	0.33	10	2273 ± 1092	4 ± 1.1	1.42 ± 0.35	0.26 ± 0.03	18.3	2273 ± 1092	4 ± 1.1	1.16 ± 0.35	0.26 ± 0.03	22.4	<0.01	2300
**273**	1.6	46	1486 ± 425	3 ± 0.3	1.48 ± 0.18	0.52 ± 0.01	35.1	1486 ± 425	3 ± 0.3	0.96 ± 0.18	0.52 ± 0.01	54.2	<0.03	10112
**233**	0.33	7.9	201.5 ± 17	4 ± 0.8	1.85 ± 0.08	0.27 ± 0.02	14.6	201.5 ± 17	4 ± 0.8	1.57 ± 0.08	0.27 ± 0.02	17.2	<0.01	7408

Estimated initial *c*-$(CH_2)_5COO$ concentration was <1 × 10^{11} molecule cm^{-3} and the fixed O_2 concentration was ~4.0 × 10^{16} molecule cm^{-3} in all measurements. The statistical uncertainties shown are 2σ.

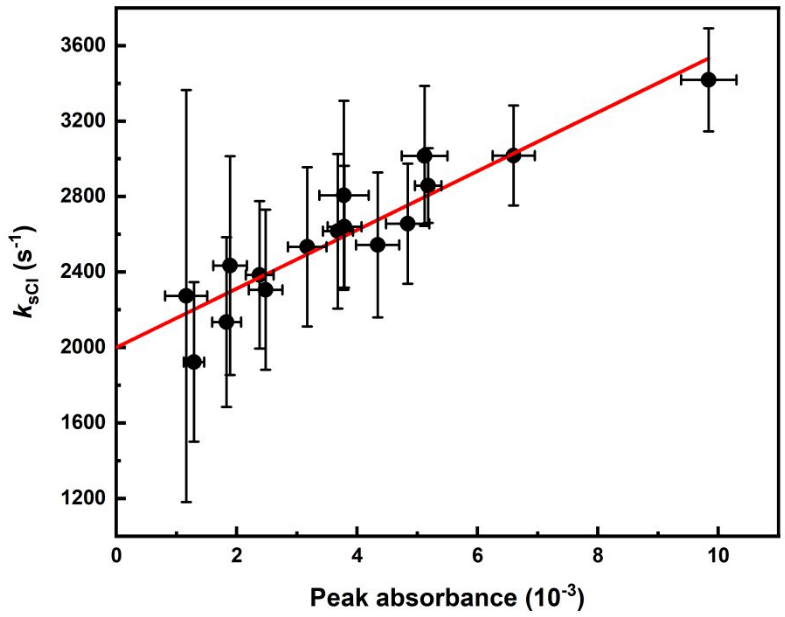

Figure S10. The obtained rate coefficients (k_{sCI}) fitted with Equation S7 to all the measured unimolecular reaction data as a function of peak absorbance of *c*-$(CH_2)_5COO$. The red line is an unweighted linear least squares fit to the data. The statistical uncertainties shown are 2σ.

The diffusion loss (k_{loss}), which originates from the diffusion out of the measurement volume, was determined by measuring the diffusion loss of CH_2OO under the same experimental conditions with the TR-BB-CEAS-apparatus. The thermal decomposition rate coefficient of CH_2OO is negligible below 375 K.[1, 10] Approximating the diffusivities of CH_2OO and *c*-$(CH_2)_5COO$ with those of formic acid (HCOOH) and 4-methylpentanoic acid ($C_6H_{12}O_2$),[11] we obtain for the k_{uni} = k_{ic}(*c*-$(CH_2)_5COO$) – $D(C_6H_{12}O_2)/D$(HCOOH) × $k_{loss,}(CH_2OO)$ = k_{ic}(*c*-$(CH_2)_5COO$) – 0.39 × $k_{loss,}(CH_2OO)$ at a given temperature and total density.

All the transient absorption traces of CH_2OO were measured at 340 nm and fitted to first-order, single exponential decay function. The initial CH_2OO concentration used in the measurements was below 2.0 × 10^{11} molecule cm^{-3}**,** which efficiently suppressed radical-radical, especially CH_2OO - CH_2OO, reactions. The values of $k_{loss}(CH_2OO)$ at each total density stay almost constant in all temperatures. Table S2 presents the obtained $k_{loss}(CH_2OO)$ values in the experimental conditions used in this work. The $k_{loss}(CH_2OO)$ values at 253–296 K were measured in our previous study.[2]

Table S2. The obtained $k_{loss}(CH_2OO)$ values in the experimental conditions used in this work. The k_{loss}(*c*-$(CH_2)_5COO$) values are calculated as 0.39 × $k_{loss}(CH_2OO)$.

T(K)	[a][N_2] (×10^{18} molecule cm^{-3})	**[b]$k_{loss}(CH_2OO)$ (s^{-1})**	**k_{loss} ($(CH_3)_2COO$) (s^{-1})**
213–296	0.33	20	8
	1.6	10	4

[a]The fixed O_2 concentration was ~4 × 10^{16} molecule cm^{-3}and the initial CH_2OO concentration was below 2.0 × 10^{11} molecule cm^{-3}. [b]The $k_{loss}(CH_2OO)$ values at 253–296 K were measured in our previous study.[2]

DFT and TST calculations

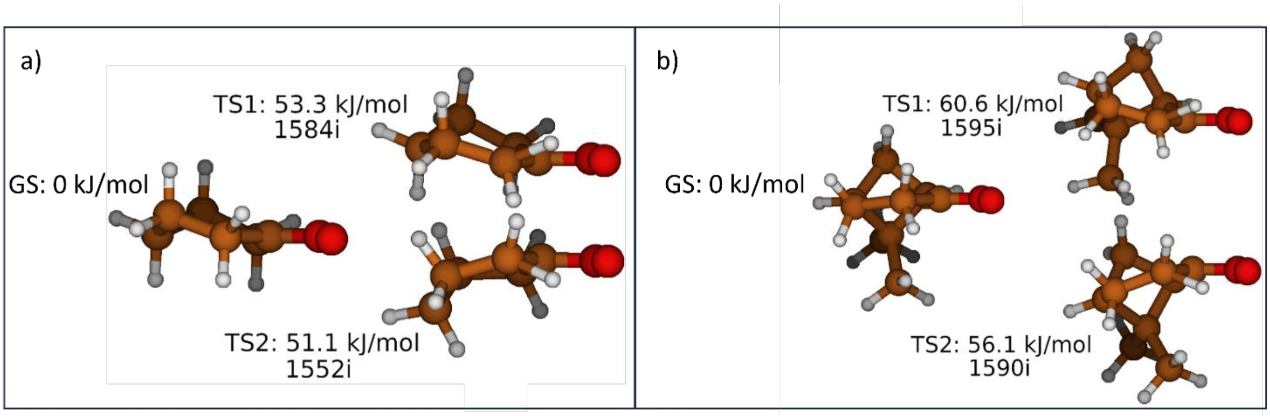

Figure S11. Relative energies and geometries of the reactants and 1,4-H-shift barriers for the (a) cyclohexanone oxide and (b) *E*-nopinone oxide systems. The zero-point-energy corrected relative energies were determined at the MN15/Def2TZVP level of theory. In both cases, two distinct 1,4-H-shift barriers were found.

To compare the 1,4-H-shift kinetics of cyclohexanone and *E*-nopinone oxides, we have performed density functional theory (DFT) calculations at the MN15/Def2TZVP[12, 13] level of theory to optimize the reactant and 1,4-H-shift saddle-point geometries, as well as to compute activation Gibbs energies ($\Delta^{‡}G$) for transition-state theory (TST) calculations. Gaussian software[14] was used to perform the MN15/Def2TZVP calculations. The activation Gibbs energies were evaluated at 298 K at the rigid-rotor-harmonic-oscillator (RRHO) level. For both oxides, the DFT calculations found two distinct 1,4-H-shift transition structures (see Fig. S11). Furthermore, all these structures have mirror images. The zero-point-energy corrected barriers for cyclohexanone oxide are, on average, about 6 kJ mol^{-1} lower for cyclohexanone oxide than *E*-nopinone oxide. The 1,4-H-shift barriers we obtain for *E*-nopinone oxide are about 10 kJ mol^{-1} lower than the CCSD(T)/aug-cc-pVTZ barriers determined by Vereecken et al.[15] Although the DFT/RRHO $\Delta^{‡}G$ values are probably not very accurate, one would still expect the relative differences between the determined $\Delta^{‡}G$ values ($\Delta\Delta^{‡}G$) to fairly accurate due to error cancellation.

To estimate how much the $\Delta^{‡}G$ differences affect the *relative* 1,4-H-shift kinetics of cyclohexanone and *E*-nopinone oxides, we applied the thermodynamic formulation of TST. The canonical (high-pressure limit) rate coefficient is calculated from

$$k = \frac{k_B T}{h} e^{-\Delta^{‡}G/RT}, \qquad \text{(S8)}$$

where k_B is the Boltzmann constant, *T* is temperature, *h* is the Planck's constant, *R* is the gas constant, and $\Delta^{‡}G$ is the Gibbs energy of activation. The room-temperature values we obtained for the Gibbs energies are $\Delta^{‡}G$(TS1) = 55.98 kJ mol^{-1} and $\Delta^{‡}G$(TS2) = 53.69 kJ mol^{-1} and $\Delta^{‡}G$(TS1) = 62.44 kJ mol^{-1} and $\Delta^{‡}G$(TS2) = 57.83 kJ mol^{-1} for the cyclohexanone oxide and *E*-nopinone oxide systems, respectively. The rate-coefficient ratio can then be estimated from

$$k_{\mathrm{r}} = \frac{k(\mathrm{TS1}) + k(\mathrm{TS2}) \text{ for cyclohexanone oxide}}{k(\mathrm{TS1}) + k(\mathrm{TS2}) \text{ for } E\text{ - nopinone oxide}} = \frac{e^{-\Delta^{‡}G(\mathrm{TS1})/RT} + e^{-\Delta^{‡}G(\mathrm{TS2})/RT} \text{ for cyclohexanone oxide}}{e^{-\Delta^{‡}G(\mathrm{TS1})/RT} + e^{-\Delta^{‡}G(\mathrm{TS2})/RT} \text{ for } E\text{ - nopinone oxide}} \qquad \text{(S9)}$$

According to this estimation, the room-temperature (298 K) 1,4-H-shift rate coefficient for cyclohexanone oxide is about 6.4 times larger than that of *E*-nopinone oxide. If the calculation is done using only the lower barriers (TS2), the ratio (k_r) is 5.3.

Tunneling was not considered in the comparison, but given the imaginary frequencies of the saddle-points are very similar, one would expect the transmission coefficients to largely cancel out. We also note that the harmonic-oscillator approximation is not very good for many of the low-frequency modes present in the structures, but again, error cancellation should ensure that the above comparison is at least semi-quantitative. The output files of the DFT calculations are available upon request.

Bimolecular reaction of *c*-$(CH_2)_5COO$ with $CF_3C(O)OH$

A dilute $CF_3C(O)OH$ reagent sample-mixture in helium for the bimolecular study was prepared in a glass bulb. A small amount of (liquid) trifluoroacetic acid (Thermo Scientific Chemicals, ≥99%) was first degassed by several freeze−pump−thaw cycles before evaporating it completely into the bulb. Two different $CF_3C(O)OH$ samples were used in the bimolecular study. The samples consisted of 0.5 and 1.16 Torr of $CF_3C(O)OH$ mixed with He in total pressure of about 1000 Torr. Dimerization of $CF_3C(O)OH$ in the bulb and the possible dimerization in the cooled reactor (at 253 K) were investigated using the available dimerization data.[16] The measured $CF_3C(O)OH$ sample pressure (p_T) in the bulb is the sum of the partial pressures of monomer (p_M) and dimer (p_D), $p_T = p_M + p_D$. The equilibrium constant, *K*, for the reaction 2 $CF_3C(O)OH \leftrightarrow (CF_3C(O)OH)_2$ can be defined as

$$K = \frac{p_D}{p_M^2} \qquad \text{(S10)}$$

From the equilibrium constant of the dimerization, the partial pressure of the monomer can be calculated as

$$p_{\mathrm{M}} = \frac{\sqrt{1+4Kp_{\mathrm{T}}}-1}{2K} \tag{S11}$$

Assuming that all the dimers fully dissociate to monomers in the reactor due to the high dilution, the total partial pressure, P_{M}, of monomers in the reactor can be expressed as by $P_{\mathrm{M}} = p_{\mathrm{M}} + 2p_{\mathrm{D}}$. By substituting $p_{\mathrm{D}} = p_{\mathrm{T}} - p_{\mathrm{M}}$, we get

$$P_{\mathrm{M}} = 2p_{\mathrm{T}} - p_{\mathrm{M}} = 2p_{\mathrm{T}} - \frac{\sqrt{1+4Kp_{\mathrm{T}}}-1}{2K} \tag{S12}$$

The equilibrium constant is 0.27 Torr $^{-1}$ and 16.2 Torr $^{-1}$ at 296 and 253 K, respectively. These values were calculated using the linearized equilibrium constant expression derived by Møgelber et al.[16]

$$\ln\left(K, \mathrm{atm}^{-1}\right) = \frac{7130.9}{T} - 18.767 \tag{S13}$$

where T is the temperature in Kelvins. Using the equilibrium value K = 0.27 Torr $^{-1}$ at 296 K, we estimated the gas-phase [$(CF_3C(O)OH)_2$] in the two different samples were 10.7 and 20% (p_{T} = 0.5 or 1.16 Torr, $p_{\mathrm{D}}/p_{\mathrm{T}} = (p_{\mathrm{T}}-p_{\mathrm{M}})/p_{\mathrm{T}}$ = 0.107 or 0.20). The dimer concentration and their dissociation have been taken into account in the given monomer concentrations (see Table S3). At 253 K (K = 16.2 Torr $^{-1}$, $p_{\mathrm{T}} \leq 1.0 \times 10^{-4}$ Torr), all dissociated dimers (high dilution) remain virtually monomers in the reactor and the low dimer concentration (< 2 ‰) is negligible in the measurements. In the main text, figure 4 shows the obtained pseudo-first-order decay coefficients of c-$(CH_2)_5COO$, $k'_{\text{c-sCI}}$, as function of [$CF_3C(O)OH$] at 253 K and 10 Torr. A bimolecular rate coefficient k(c-$(CH_2)_5COO$ + $CF_3C(O)OH$) is obtained from the slope of the equation $k'_{\text{c-sCI}} = k_{\text{loss}}$ + k(c-$(CH_2)_5COO$ + $CF_3C(O)OH$) × [$CF_3C(O)OH$] fitted to the data, while the intercept reflects the k_{loss}. The complete results are shown in Table S3 along with the experimental conditions.

Table S3 Experimental results and conditions used to determine the bimolecular rate coefficient of (8.7±1.0) × 10^{-10} cm^3 $molecule^{-1}$ s^{-1} for the c-$(CH_2)_5COO$ + $CF_3C(O)OH$ reaction at 253 K and 10 Torr[a].

Day #	$CF_3C(O)OH$ sample #[b]	[$CF_3C(O)OH$] ($\times 10^{12}$ molecule cm^{-3})	$k'_{\text{c-sCI}}$ (s^{-1})[c]	k_{NR} (s^{-1})[c]	A_{sCI} ($\times 10^{-3}$)[c]	A_{NR} ($\times 10^{-3}$)[c]	A_{NR} / A_{sCI} (%)	Number of averages
1	1	0	613 ± 74	8 ± 0.8	4.51 ± 0.19	1.06 ± 0.03	23.8	3018
1	1	1.33	1650 ± 293	7 ± 1.1	3.94 ± 0.29	0.64 ± 0.02	16.1	3494
1	1	2.65	2408 ± 546	3 ± 1.1	3.94 ± 0.37	0.60 ± 0.02	15.2	2757
1	1	3.97	3753 ± 904	3 ± 0.4	3.70 ± 0.36	0.63 ± 0.02	17.1	4193
2	2	0	542 ± 70	8 ± 0.9	3.98 ± 0.18	0.96 ± 0.03	24.2	2596
2	2	1.31	1566 ± 264	6 ± 1.3	3.67 ± 0.26	0.50 ± 0.02	13.5	3379
2	2	2.35	2188 ± 477	5 ± 1.3	3.33 ± 0.31	0.47 ± 0.02	14.1	3500
2	2	3.12	3358 ± 863	5 ± 1.2	3.67 ± 0.39	0.54 ± 0.02	14.6	2975
2	2	3.89	4381 ± 1183	6 ± 1.4	3.80 ± 0.44	0.49 ± 0.02	12.9	3011
3	2	0	499 ± 77	7 ± 1.1	3.56 ± 0.19	0.81 ± 0.03	22.7	2039
3	2	1.83	1850 ± 345	4 ± 1.3	2.85 ± 0.23	0.36 ± 0.02	12.8	5043
3	2	2.86	3111 ± 707	5 ± 1.4	2.82 ± 0.27	0.36 ± 0.02	12.7	5616
3	2	3.63	3549 ± 719	5 ± 1.1	2.93 ± 0.25	0.40 ± 0.01	13.6	7753

[a]The total number density at 253 K and 10 Torr was 3.8 × 10^{17} molecule cm^{-3}. Estimated initial c-$(CH_2)_5COO$ concentration was <5.0 × 10^{10} molecule cm^{-3} and the fixed O_2 concentration was ~4.0 × 10^{16} molecule cm^{-3} for all measurements. [b]The $CF_3C(O)OH$ reactant concentration in the reactor is calculated with accounting for the dimerization of the dilute $CF_3C(O)OH$ in helium mixture prepared in a bulb.[16] The relative [$CF_3C(O)OH$] of the sample #1 and sample #2 were 1.39 Torr/1001.5 Torr and 0.555 Torr/1008.8 Torr. [c]The statistical uncertainties shown are 2σ.

References

(1) Peltola, J.; Seal, P.; Inkilä, A.; Eskola, A. Time-resolved, broadband UV-absorption spectrometry measurements of Criegee intermediate kinetics using a new photolytic precursor: unimolecular decomposition of CH_2OO and its reaction with formic acid. *Phys. Chem. Chem. Phys.* **2020**, *22*, 11797-11808.

(2) Peltola, J.; Seal, P.; Vuorio, N.; Heinonen, P.; Eskola, A. Solving the discrepancy between the direct and relative-rate determinations of unimolecular reaction kinetics of dimethyl-substituted Criegee intermediate $(CH_3)_2COO$ using a new photolytic precursor. *Phys. Chem. Chem. Phys.* **2022**, *24*, 5211-5219.

(3) Franzon, L.; Peltola, J.; Valiev, R.; Vuorio, N.; Kurtén, T.; Eskola, A. An experimental and master equation investigation of kinetics of the CH_2OO + RCN reactions (R= H, CH_3, C_2H_5) and their atmospheric relevance. *J. Phys. Chem. A* **2023**, *127*, 477-488.

(4) Chang, Y.-P.; Chang, C.-H.; Takahashi, K. Absolute UV absorption cross sections of dimethyl substituted Criegee intermediate $(CH_3)_2COO$. *Chem. Phys. Lett.* **2016**, *653*, 155-160.

(5) Liu, F.; Beames, J. M.; Green, A. M.; Lester, M. I. UV spectroscopic characterization of dimethyl-and ethyl-substituted carbonyl oxides. *J. Phys. Chem. A* **2014**, *118*, 2298-2306.

(6) Westley, E.; Sowden, M. J.; Magann, N. L.; Horvath, K. L.; Connor, K. P.; Sherburn, M. S. Substituted tetraethynylethylene–tetravinylethylene hybrids. *J. Am. Chem. Soc.* **2022**, *144*, 977-986.

(7) Ting, W.-L.; Chang, C.-H.; Lee, Y.-F.; Matsui, H.; Lee, Y.-P.; Lin, J. J.-M. Detailed mechanism of the CH_2I + O_2 reaction: Yield and self-reaction of the simplest Criegee intermediate CH_2OO. *J. Chem. Phys.* **2014**, *141*, 1-11.

(8) Huang, H.-L.; Chao, W.; Lin, J. J.-M. Kinetics of a Criegee intermediate that would survive high humidity and may oxidize atmospheric SO_2. *Proc. Natl. Acad. Sci. U.S.A.* **2015**, *112*, 10857-10862.

(9) Smith, M. C.; Chao, W.; Takahashi, K.; Boering, K. A.; Lin, J. J.-M. Unimolecular decomposition rate of the Criegee intermediate $(CH_3)_2COO$ measured directly with UV absorption spectroscopy. *J. Phys. Chem. A* **2016**, *120*, 4789-4798.

(10) Stone, D.; Au, K.; Sime, S.; Medeiros, D. J.; Blitz, M.; Seakins, P. W.; Decker, Z.; Sheps, L. Unimolecular decomposition kinetics of the stabilised Criegee intermediates CH_2OO and CD_2OO. *Phys. Chem. Chem. Phys.* **2018**, *20*, 24940-24954.

(11) Tang, M.; Shiraiwa, M.; Pöschl, U.; Cox, R.; Kalberer, M. Compilation and evaluation of gas phase diffusion coefficients of reactive trace gases in the atmosphere: Volume 2. Diffusivities of organic compounds, pressure-normalised mean free paths, and average Knudsen numbers for gas uptake calculations. *Atmos. Chem. Phys.* **2015**, *15*, 5585-5598.

(12) Haoyu, S. Y.; He, X.; Li, S. L.; Truhlar, D. G. MN15: A Kohn–Sham global-hybrid exchange–correlation density functional with broad accuracy for multi-reference and single-reference systems and noncovalent interactions. *Chemical science* **2016**, *7*, 5032-5051.

(13) Weigend, F.; Ahlrichs, R. Balanced basis sets of split valence, triple zeta valence and quadruple zeta valence quality for H to Rn: Design and assessment of accuracy. *Phys. Chem. Chem. Phys.* **2005**, *7*, 3297-3305.

(14) Frisch, M. e.; Trucks, G.; Schlegel, H. B.; Scuseria, G.; Robb, M.; Cheeseman, J.; Scalmani, G.; Barone, V.; Petersson, G.; Nakatsuji, H. Gaussian 16. Gaussian, Inc. Wallingford, CT: 2016.

(15) Vereecken, L.; Novelli, A.; Taraborrelli, D. Unimolecular decay strongly limits the atmospheric impact of Criegee intermediates. *Phys. Chem. Chem. Phys.* **2017**, *19*, 31599-31612.

(16) Møgelberg, T. E.; Nielsen, O. J.; Sehested, J.; Wallington, T. J.; Hurley, M. D. Atmospheric chemistry of CF_3COOH. Kinetics of the reaction with OH radicals. *Chem. Phys. Lett.* **1994**, *226*, 171-177.
