# Supplementary material for: Direct Kinetic Measurements of a Cyclic Criegee Intermediate; Unimolecular Decomposition of c-(CH2)5COO
Source: J Phys Chem Lett. 2024 May 10;15(20):5331–6. doi: 10.1021/acs.jpclett.4c00554 (PMC11389976; doi:10.1021/acs.jpclett.4c00554)
Supplement: Supplementary file 2 — jz4c00554_si_002.pdf [file jz4c00554_si_002.pdf]

jz-2024-00554e.R1

Name: Peer Review Information for "Direct Kinetic Measurements of a Cyclic Criegee Intermediate; Unimolecular Decomposition of <i>c</i>-(CH₂)₅COO"

First Round of Reviewer Comments

Reviewer: 1

Comments to the Author

Review Comments

Journal: The Journal of Physical Chemistry Letters
Manuscript ID: jz-2024-00554e
Original Submission Date: 21-Feb-2024
Title : "Direct Kinetic Measurements of a Cyclic Criegee Intermediate; Unimolecular Decomposition of <i>c</i>-(CH₂)₅ COO" Author(s): Peltola, Jari; Heinonen, Petri; Eskola, Arkke

This paper reports the kinetic measurements of the thermal unimolecular decomposition of the Criegee intermediate *c*-$(CH_2)_5COO$ and its reaction with $CF_3C(O)OH$ by using the cavity enhanced UV absorption technique. The authors synthesized the precursor *c*-$(CH_2)_5CIBr$ and photolyzed at 213 nm in the presence of O_2 to produce *c*-$(CH_2)_5COO$, which was probed at 340 nm. The reported unimolecular decomposition rate coefficient of 1998 ± 147 s^{-1} at 296 K is more than 5 times greater than the theoretical prediction. The rate coefficient of *c*-$(CH_2)_5COO$ + $CF_3C(O)OH$ at 253 K and 10 Torr, $(8.7 \pm 1.0) \times 10^{-10}$ cm^3 $molecule^{-1}$ s^{-1}, is similar to those of the Criegee intermediate CH_2OO with $CF_3C(O)OH$.

1. The major advance of this work is, following the authors' previous ideas, the use of a precursor *c*-$(CH_2)_5CIBr$ instead of *c*-$(CH_2)_5CI_2$, which is highly unstable. The slight disadvantage of this method is that a shorter wavelength (in this case 213 nm) for photolysis is required. This concept can be applied to other systems as well and will be useful when the $R_1R_2CI_2$ precursor is unstable. However, one shortcoming of this paper is that the authors did not report the characterization of this preparation method in detail. For example, what are the yield of the Criegee intermediates and what are the coproducts? I would imagine that *c*$(CH_2)_5CIOO$, *c*-$(CH_2)_5CBrOO$, and other open-chain byproducts might be present in the system and interfere.

2. The authors reported a fast unimolecular decay coefficient from a simplified analysis by fitting the data with single exponential decays. In the text, they stated that "The first term in Equation (1) represents the fast decay of *c*-$(CH_2)_5COO$, while the second term describes the slow decay of nonreactive species that are formed at the same rate (k_{sCI}) as *c*-$(CH_2)_5COO$ decays." Because Eq. 1 was missing in the text that I received, I cannot judge the correctness of Eq. 1. A single exponential decay of a nonreactive species formed at time zero (Eq. S7) is quite different from a slow decay of nonreactive species that were formed at the same rate (k_{sCI}) as *c*-$(CH_2)_5COO$ decays. The latter will lead to an increased decay rate coefficient of the Criegee intermediates when its concentration is high. I do not believe that "Both fitting models returned essentially the same values for k_{sCI}, with a difference < 0.01%", as claimed by these authors. The authors should list the results of both fittings in Table S2 to compare. Also, Table S2 seems to cover only the four colored points in Figure 2, not the black points.

Finally, in Figures S8 and S9, the time scale was not small enough to show clearly the decay of the absorbance and the fitted curves.

3. No discussion on the large discrepancy between theoretically predicted k_{SCI} and experimental values were presented. If only the data in Figure 2 for absorbance less than 7×10^{-3} were used in fitting, the intercept might be greatly reduced.

4. No error analysis except statistical errors in fitting was provided. For example, the error in Figure 2. With k near 4000 s^{-1}, the number of data points at 67-μs intervals used in their experiments might not be enough to accurately describe the decay and cause errors. The errors in using the simplified model should also be discussed.

I recommend publication of this manuscript after the authors considered the above comments and revised the manuscript accordingly.

Minor points:

1. Page 6, "The transient absorption signal of *c*-$(CH_2)_5COO$ mainly contains contributions from the thermal unimolecular reaction and the self-reaction," The "signal" cannot have contributions from "reactions"; perhaps the authors meant the decay of the signal.
2. Page 11, TR-BB-CEAS needs a full name first.
3. In abstract and elsewhere, 1998 ± 147 seems to carry too many significant digits.

Reviewer: 2

Comments to the Author

The authors provide the first direct measurement of the rate of unimolecular decomposition for a cyclic Criegee intermediate. These results are important to our understanding of the role of atmospherically relevant Criegee intermediates. The conclusions are well supported by the data, and the methodology is well presented. Thus, I consider this manuscript highly appropriate for

publication in J. Phys. Chem. Lett. I have only a few suggestions of places where the wording and/or discussion could be slightly improved.

Most significantly, the statement on p. 11 that the cyclobutyl radical is a mere spectator is probably overstated. I can certainly see that it is somewhat distant from the interaction site, but is that distance enough to remove all chemical effects on the interactions. It is not obvious to me that it is. A simple DFT calculation of the barrier heights for the two cases would have helped to validate their assumption.

On p. 3, the authors state "Only recently discovered HOMs contribute …" This sentence reads as though other earlier discovered HOMs don't contribute to the formation of SOAs. Whereas, what I believe they intend is that "HOMs, which have only recently been discovered, contribute to the formation of SOAs" or perhaps the "The importance of HOMS to the formation of SOAs has only recently been discovered."

On p. 4, the authors state "They are also less reactive with sCIs and appear to be resistant to secondary chemistry as they do not produce, for example, R1R2CI radicals (and consequently R1R2COO) in X + R1R2CIBr → XI + R1R2CBr reaction, where X is any species" I am not able to decipher what the authors intended with this sentence. The reaction is making the Bromine radical, but the statement is about not making the Iodine radical. Why did they switch between the two radicals. Also, is there any citation that could be provided for this statement of the lack of X + R1R2CIBr reaction. And, are they really saying that no X can abstract from R1R2CBr?

On p. 5, the authors use the label CF3COOH to denote TFA. I know others use this sort of notation, but I consider it to be nonchemical and problematic as it does not properly denote the chemical bonding. I see that label and I immediately think you have some sort of carbene hydroperoxide. I would much prefer something like CF3C(O)OH.

On p. 6 and 12, the authors talk about exceeding the gas kinetic limit. Again, I know others use that expression, but it is, of course, physically impossible for a reaction to exceed the gas kinetic limit – how do you have a reaction without a collision? What is really meant is that the rate exceeds some sort of crude hard-sphere calculated gas kinetic limit. Notably, it essentially trivial to calculate and compare (and much more valuable) the rate with a long-range transition state theory expression that properly incorporates dipole-dipole (and other long-range interactions) and which would be much more representative of the true gas kinetic limit.

Author's Response to Peer Review Comments:

Our answers to the referees, including changes and additions made to the manuscript and SI, are explained in the file attached below. We have also made the requested non-scientific changes to the manuscript.

The Journal of Physical Chemistry Letters
Manuscript ID: jz-2024-00554e

TITLE: Direct Kinetic Measurements of a Cyclic Criegee Intermediate; Unimolecular Decomposition of *c*-$(CH_2)_5COO$

Jari Peltola, Petri Heinonen and Arkke Eskola*

REVISED MANUSCRIPT AND RESPONSE TO THE REVIEWERS' COMMENTS

We thank the reviewers' for their comments to improve the manuscript. We have revised the manuscript and supporting information based on their comments. Please notice the following response to the reviewers' comments. The page numbers refer to the revised manuscript and supporting information.

Reviewer: 1

Comments:

This paper reports the kinetic measurements of the thermal unimolecular decomposition of the Criegee intermediate c-$(CH_2)_5COO$ and its reaction with $CF_3C(O)OH$ by using the cavity enhanced UV absorption technique. The authors synthesized the precursor c-$(CH_2)_5ClBr$ and photolyzed at 213 nm in the presence of O_2 to produce c-$(CH_2)_5COO$, which was probed at 340 nm. The reported unimolecular decomposition rate coefficient of 1998 ± 147 s^{-1} at 296 K is more than 5 times greater than the theoretical prediction. The rate coefficient of c-$(CH_2)_5COO$ + $CF_3C(O)OH$ at 253 K and 10 Torr, (8.7 ± 1.0) × 10^{-10} cm^3 $molecule^{-1}$ s^{-1}, is similar to those of the Criegee intermediate CH_2OO with $CF_3C(O)OH$.

1. The major advance of this work is, following the authors' previous ideas, the use of a precursor c$(CH_2)_5ClBr$ instead of c-$(CH_2)_5Cl_2$, which is highly unstable. The slight disadvantage of this method is that a shorter wavelength (in this case 213 nm) for photolysis is required. This concept can be applied to other systems as well and will be useful when the $R_1R_2CI_2$ precursor is unstable. However, one shortcoming of this paper is that the authors did not report the characterization of this preparation method in detail. For example, what are the yield of the Criegee intermediates and what are the coproducts? I would imagine

that c-$(CH_2)_5$ClOO, c-$(CH_2)_5$CBrOO, and other open-chain byproducts might be present in the system and interfere.

The absorption cross-sections of c-$(CH_2)_5$COO and c-$(CH_2)_5$ClBr are needed to determine the yield of the cyclic Criegee intermediate. Since they have not been measured, we feel that the determination of the Criegee yield is not within the scope of this manuscript (letter). In addition, the measurement of the *c*-$(CH_2)_5$ClBr cross-section would be difficult to perform with the current experimental setup because its vapor pressure is very low (<<1 Torr), making it difficult to determine its exact concentration.

In the Support Information on page S8, we discuss that the observed additional absorption (offset) in the experimental signal, which increases with increasing pressure, may originate from an enhanced stabilization of the *c*-$(CH_2)_5$ClOO radical. Other byproducts can also be present in the system, but the characterization in the UV-region is difficult with the current experimental setup.

2. The authors reported a fast unimolecular decay coefficient from a simplified analysis by fitting the data with single exponential decays. In the text, they stated that “The first term in Equation (1) represents the fast decay of c-$(CH_2)_5$COO, while the second term describes the slow decay of nonreactive species that are formed at the same rate (k_{sCI}) as c-$(CH_2)_5$COO decays.” Because Eq. 1 was missing in the text that I received, I cannot judge the correctness of Eq. 1. A single exponential decay of a nonreactive species formed at time zero (Eq. S7) is quite different from a slow decay of nonreactive species that were formed at the same rate (k_{sCI}) as c-$(CH_2)_5$COO decays. The latter will lead to an increased decay rate coefficient of the Criegee intermediates when its concentration is high. I do not believe that “Both fitting models returned essentially the same values for k_{sCI}, with a difference < 0.01%”, as claimed by these authors. The authors should list the results of both fittings in Table S2 to compare. Also, Table S2 seems to cover only the four colored points in Figure 2, not the black points. Finally, in Figures S8 and S9, the time scale was not small enough to show clearly the decay of the absorbance and the fitted curves.

The Equation (1) is (and was) presented in the Experimental Methods on page 14 of the manuscript. As the reviewer suggested, we have now added the results of both fittings of the full data (presented in Fig. 2) in Table S2 (page S10) for comparison. All the fittings presented in Table S2 have been refitted and reviewed. The comparison shows, with a few exceptions, that the obtained k_{sCI} values are identical to the accuracy of the four displayed numbers, regardless of the fitting method. Overall, the deviation was less than 0.05%. The only significant difference is the obtained lower peak absorbance (A_{sCI}) of the Criegee intermediate when using Equation S7. The intercept of the unweighted linear least squares fit to the rate coefficients (k_{sCI}) obtained by Equation S7 as a function of peak absorbance of *c*-$(CH_2)_5$COO is 2000 ± 144 s^{-1} (see figure S10), which is essentially the same value than when using Eq. 1 (2006 ± 147 s^{-1}). In conclusion, there is no significant difference between the obtained kinetic results using different fitting models.

We have added the revised comparison of the fitting models to page S8 and S10 of the Supplement Information. In addition, the figures S8 and S9 have now been modified so that they show the fitted curves on a shorter timescale and the insets show the entire signal.

3. No discussion on the large discrepancy between theoretically predicted k_{SCI} and experimental values were presented. If only the data in Figure 2 for absorbance less than 7×10^{-3} were used in fitting, the intercept might be greatly reduced.

To compare the 1,4-H-shift kinetics of cyclohexanone and *E*-nopinone oxides, we have performed density functional theory (DFT) calculations at the MN15/Def2TZVP level of theory to optimize the reactant and 1,4-H-shift saddle-point geometries, as well as to compute activation Gibbs energies ($\Delta^{\ddagger}G$). The activation Gibbs energies were evaluated at 298 K at the rigid-rotor-harmonic-oscillator (RRHO) level. For both oxides, the DFT calculations found two distinct 1,4-H-shift transition structures. Furthermore, all these structures have mirror images. The 1,4-H-shift barriers are, on average, about 6 kJ mol^{-1} lower for cyclohexanone oxide than *E*-nopinone oxide. By applying the thermodynamic formulation of transition-state theory (see Supporting Information for details), we find the unimolecular decay of cyclohexanone oxide to be about 5–6 faster than that of *E*-nopinone oxide. This finding is consistent with the k_{uni}(c-$(CH_2)_5$COO) = 1998 ± 147 s^{-1} value measured in this study (296 K) for cyclohexanone oxide and the recently calculated value of 375 s^{-1} for *E*-nopinone oxide. Note that although the individual DFT/RRHO $\Delta^{\ddagger}G$ values are probably not very accurate, we expect the errors to be fairly systematic and cancel out in the comparison. That is to say, the $\Delta\Delta^{\ddagger}G$ values that were used to do the comparison should be more accurate than the individual $\Delta^{\ddagger}G$ values. Tunneling was not considered, but given the imaginary frequencies of the saddle-points are very similar, one would expect the transmission coefficients to largely cancel out in the comparison.

We have added the following discussion in the second column on page 11: "To compare the 1,4-Hatom-shift kinetics of cyclohexanone oxide and *E*-nopinone oxide, we performed density functional theory (DFT) calculations at the MN15/Def2TZVP level of theory for both systems to compute activation Gibbs energies for the 1,4-H-atom-shift transition states. The computed activation Gibbs energies are about 5 kJ mol^{-1} lower for the cyclohexanone oxide system. Because of the massive tunnelling effect, the activation energy obtained from the Arrhenius fit is about a factor of two smaller than the zero-point-energy corrected 1,4-H-shift barrier heights. By applying the thermodynamic formulation of transition state theory (TST), we estimate that the cyclohexanone oxide system has a 5–6 times larger 1,4-H-atom-shift rate coefficient than the *E*-nopinone oxide system. This simple estimate of the reactivity difference compares well with the experimental results obtained in the current study and the room-temperature computation by Vereecken et al.[6] At 296 K and close to the high-pressure limit, we measured k_{uni}(*c*-$(CH_2)_5$COO) = 1998 ± 147 s^{-1} for cyclohexanone oxide, whereas Vereecken et al.[6] obtained 375 s^{-1} for *E*-nopinone oxide. The DFT/TST calculations are explained in more detail in the Supporting Information. "

In addition, when the two highest absorbances are omitted from the data in Fig. 2, the intercept (k_{IC}) is 1896 ± 200 s^{-1} (see the figure below), which is only about 100 s^{-1} less than when using the full data (2006 ± 147 s^{-1}). Since the results are consistent within the uncertainties, there is no significant difference between the results when using different data.

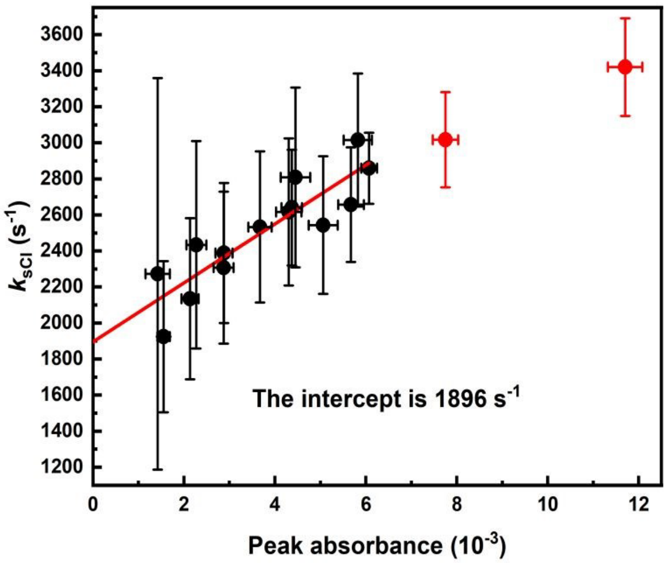

An alternative unweighted linear least-squares fit to the data in Fig 2. with the two highest absorbances omitted.

4. No error analysis except statistical errors in fitting was provided. For example, the error in Figure 2. With k near 4000 s^{-1}, the number of data points at 67-μs intervals used in their experiments might not be enough to accurately describe the decay and cause errors. The errors in using the simplified model should also be discussed.

We have added to the end of the Experimental methods section—starting on page 14—the following "The statistical fitting uncertainties shown in this study are 2σ. This includes uncertainties of all the measured exponential decays (k_{sCI} and $k'_{c\text{-sCI}}$) and linear least squares fits. The estimated overall uncertainty in the reported rate-coefficient values is ±20%."

We have also added the following to page S8 of the Supporting Information: ''Although a more accurate kinetics model would give better fits to the absorption traces, the intercept values of the more accurate and simplified models would be almost the same since the reactive species are formed at small concentrations. These concentrations are proportional to [*c*-$(CH_2)_5CIBr$]$_0$ and the laser pulse energy. Smith et al.[9] have stated this previously in their study.''

Minor points:

1. Page 6, "The transient absorption signal of c-$(CH_2)_5$COO mainly contains contributions from the thermal unimolecular reaction and the self-reaction," The "signal" cannot have contributions from "reactions"; perhaps the authors meant the decay of the signal.

This has been corrected. It says now (on page 6) "The decay of the c-$(CH_2)_5$COO absorption signal mainly...''

2. Page 11, TR-BB-CEAS needs a full name first.

Corrected. The unnecessary abbreviation was completely removed from the manuscript.

3. In abstract and elsewhere, 1998 ± 147 seems to carry too many significant digits.

Since the diffusion loss (k_{loss}), which is subtracted from the experimentally obtained k_{ic}-values to determine the unimolecular rate coefficient, is small (<10 s^{-1}), we think that reporting the k_{uni}-values to three significant digits leads to a loss of information.

Reviewer: 2

Comments:

The authors provide the first direct measurement of the rate of unimolecular decomposition for a cyclic Criegee intermediate. These results are important to our understanding of the role of atmospherically relevant Criegee intermediates. The conclusions are well supported by the data, and the methodology is well presented. Thus, I consider this manuscript highly appropriate for publication in J. Phys. Chem. Lett. I have only a few suggestions of places where the wording and/or discussion could be slightly improved.

Most significantly, the statement on p. 11 that the cyclobutyl radical is a mere spectator is probably overstated. I can certainly see that it is somewhat distant from the interaction site but is that distance enough to remove all chemical effects on the interactions. It is not obvious to me that it is. A simple DFT calculation of the barrier heights for the two cases would have helped to validate their assumption.

To compare the 1,4-H-shift kinetics of cyclohexanone and *E*-nopinone oxides, we have performed density functional theory (DFT) calculations at the MN15/Def2TZVP level of theory to optimize the reactant and 1,4-H-shift saddle-point geometries, as well as to compute activation Gibbs energies ($\Delta^{\ddagger}G$). The activation Gibbs energies were evaluated at 298 K at the rigid-rotor-harmonic-oscillator (RRHO) level. For both oxides, the DFT calculations found two distinct 1,4-H-shift transition structures. Furthermore, all these structures have mirror images. The 1,4-H-shift barriers are, on average, about 6 kJ mol^{-1} lower for cyclohexanone oxide than *E*-nopinone oxide. By applying the thermodynamic formulation of transition-state theory (see Supporting Information for details), we find the unimolecular decay of cyclohexanone oxide to be about 5–6 faster than that of *E*-nopinone oxide. This finding is consistent with the k_{uni}(c-$(CH_2)_5$COO) = 1998 ± 147 s^{-1} value measured in this study (296 K) for cyclohexanone oxide and the recently calculated value of 375 s^{-1} for *E*-nopinone oxide. Note that although the individual DFT/RRHO $\Delta^{\ddagger}G$ values are probably not very accurate, we expect the errors to be fairly systematic and cancel out in the comparison. That is to say, the $\Delta\Delta^{\ddagger}G$ values that were used to do the comparison should be more accurate than the individual $\Delta^{\ddagger}G$ values. Tunneling was not considered, but given the imaginary frequencies of the saddle-points are very similar, one would expect the transmission coefficients to largely cancel out in the comparison.

We have added the following discussion in the second column on page 11: "To compare the 1,4-Hatom-shift kinetics of cyclohexanone oxide and *E*-nopinone oxide, we performed density functional theory (DFT) calculations at the MN15/Def2TZVP level of theory for both systems to compute activation Gibbs energies for the 1,4-H-atom-shift transition states. The computed activation Gibbs energies are about 5 kJ mol^{-1} lower for the cyclohexanone oxide system. Because of the massive tunnelling effect, the activation energy obtained from the Arrhenius fit is about a factor of two smaller than the zero-point-energy corrected 1,4-H-shift barrier heights. By applying the thermodynamic formulation of transition state theory (TST), we estimate that the cyclohexanone oxide system has a 5–6 times larger 1,4-H-atom-shift rate coefficient than the *E*-nopinone oxide system. This simple estimate of the reactivity difference compares well with the experimental results obtained in the current study and the room-temperature computation by Vereecken et al.[6] At 296 K and close to the high-pressure limit, we measured $k_{uni}(c\text{-}(CH_2)_5COO)$ = 1998 ± 147 s^{-1} for cyclohexanone oxide, whereas Vereecken et al.[6] obtained 375 s^{-1} for *E*-nopinone oxide. The DFT/TST calculations are explained in more detail in the Supporting Information."

On p. 3, the authors state "Only recently discovered HOMs contribute …" This sentence reads as though other earlier discovered HOMs don't contribute to the formation of SOAs. Whereas, what I believe they intend is that "HOMs, which have only recently been discovered, contribute to the formation of SOAs" or perhaps the "The importance of HOMS to the formation of SOAs has only recently been discovered."

This has been clarified. It says now (on page 3) "These recently discovered HOMs, formed via terpene autoxidation involving peroxyl radicals, contribute to the formation of secondary organic aerosols (SOAs),…"

On p. 4, the authors state "They are also less reactive with sCIs and appear to be resistant to secondary chemistry as they do not produce, for example, R_1R_2CI radicals (and consequently R_1R_2COO) in X + R_1R_2CIBr → XI + R_1R_2CBr reaction, where X is any species" I am not able to decipher what the authors intended with this sentence. The reaction is making the Bromine radical, but the statement is about not making the Iodine radical. Why did they switch between the two radicals. Also, is there any citation that could be provided for this statement of the lack of X + R_1R_2CIBr reaction. And, are they really saying that no X can abstract from R_1R_2CBr?

Our intention here is to say that the X + R_1R_2CIBr reaction, where X is any species, is more likely to produce the R_1R_2CBr radical than the R_1R_2CI radical. Since the R_1R_2CBr + O_2 reaction does not produce R_1R_2COO, no additional R_1R_2COO is produced in the system.

We have reviewed the sentence. It says now on page 4: "They are also less reactive with sCIs and appear to be resistant to secondary chemistry, since the X + R_1R_2CIBr reaction, where X is any species, is more likely to produce the R_1R_2CBr radical (+ XI) than the R_1R_2CI radical (+ XBr).[11] Thus, no additional R_1R_2COO is produced because the R_1R_2CBr + O_2 reaction does not produce R_1R_2COO." *On p. 5, the authors use the label CF_3COOH to denote TFA. I know others use this sort of notation, but I consider it to be nonchemical and problematic as it does not properly denote the chemical*

bonding. I see that label and I immediately think you have some sort of carbene hydroperoxide. I would much prefer something like $CF_3C(O)OH$.

The notation CF_3COOH has been changed to $CF_3C(O)OH$.

On p. 6 and 12, the authors talk about exceeding the gas kinetic limit. Again, I know others use that expression, but it is, of course, physically impossible for a reaction to exceed the gas kinetic limit – how do you have a reaction without a collision? What is really meant is that the rate exceeds some sort of crude hard-sphere calculated gas kinetic limit. Notably, it essentially trivial to calculate and compare (and much more valuable) the rate with a long-range transition state theory expression that properly incorporates dipole-dipole (and other long-range interactions) and which would be much more representative of the true gas kinetic limit.

We agree that claiming that the rate exceeds the gas kinetic limit is somewhat misleading. It was used to emphasize that Criegee intermediate + acid reactions are extremely fast. We have removed this comparison with the gas kinetic limit from the revised manuscript.
